# Modeling the Cellular Mechanisms and Olfactory Input Underlying the Triphasic Response of Moth Pheromone-Sensitive Projection Neurons

**DOI:** 10.1371/journal.pone.0126305

**Published:** 2015-05-11

**Authors:** Yuqiao Gu

**Affiliations:** 1 UMR 1392 iEES-Paris, National Institute for Agricultural Research, Versailles, France; 2 Cognitive Neuroscience, International School for Advanced Studies, Trieste, Italy; Claremont Colleges, UNITED STATES

## Abstract

In the antennal lobe of the noctuid moth *Agrotis ipsilon*, most pheromone-sensitive projection neurons (PNs) exhibit a triphasic firing pattern of excitation (E_1_)-inhibition (I)-excitation (E_2_) in response to a pulse of the sex pheromone. To understand the mechanisms underlying this stereotypical discharge, we developed a biophysical model of a PN receiving inputs from olfactory receptor neurons (ORNs) via nicotinic cholinergic synapses. The ORN is modeled as an inhomogeneous Poisson process whose firing rate is a function of time and is fitted to extracellular data recorded in response to pheromone stimulations at various concentrations and durations. The PN model is based on the Hodgkin-Huxley formalism with realistic ionic currents whose parameters were derived from previous studies. Simulations revealed that the inhibitory phase I can be produced by a SK current (Ca^2+^-gated small conductance K^+^ current) and that the excitatory phase E_2_ can result from the long-lasting response of the ORNs. Parameter analysis further revealed that the ending time of E_1_ depends on some parameters of SK, Ca^2+^, nACh and Na^+^ currents; I duration mainly depends on the time constant of intracellular Ca^2+^ dynamics, conductance of Ca^2+^ currents and some parameters of nACh currents; The mean firing frequency of E_1_ and E_2_ depends differentially on the interaction of various currents. Thus it is likely that the interplay between PN intrinsic currents and feedforward synaptic currents are sufficient to generate the triphasic firing patterns observed in the noctuid moth *A*. *ipsilon*.

## Introduction

Odor coding by the olfactory system has been studied by various experimental and modeling approaches. Natural odor stimuli can be characterized not only by their molecular features but also by properties such as concentration, spatial and temporal change of chemical components. Behavioral experiments on vertebrates [1], terrestrial [2–5] and aquatic invertebrates [6–7] showed that the physical characteristics of odor stimuli condition the behavioral response to an odorant. In moths, for example, intermittent and continuous stimulation with the same odor (the sex pheromone) evokes two distinct flight behaviors of upwind zig-zagging flight towards the odor source and cast/counter turn across the wind line, respectively [8]. The stimulation features are encoded and analyzed by individual neurons and neural networks of the olfactory system involving the antennae, the antennal lobes (ALs), the mushroom bodies (MBs) and the lateral horn in insects. Odorants are first detected and encoded by different types of olfactory receptor neurons (ORNs) situated in the antenna. Features of odorant stimuli are further analyzed in the AL, the first-order processing center. ORNs of the same type project to the same glomerulus [9] where they establish synaptic connections with multiglomerular local neurons (LNs), intrinsic to the AL, and uniglomerular projection neurons (PNs) [9]. The stimulation features/parameters influence the spatial and temporal activity patterns throughout the glomerular array and the response characteristics of individual PNs [10]. However, the neural basis of the differential response to the different physical odor stimulation features of the same odorant is poorly known. Neurons at different processing stages show different response patterns in response to the same stimulus [11–13]. Recordings in mitral/tufted cells in vertebrates and PNs in insects revealed that the odor-evoked responses of these second-order neurons are generally complex, consisting of both depolarizing and hyperpolarizing phases [12–19]. Remarkably, the temporal patterns of spike activity observed in some vertebrate mitral/tufted cells and insect PNs are very similar [12–20], suggesting common principles of cellular and/or synaptic mechanisms. In the macroglomerular complex (MGC) of the moth *A*. *ipsilon*, i.e. the specialist system processing pheromone information in the insect AL, a large majority of pheromone-sensitive PNs exhibit a triphasic firing pattern when the antenna is stimulated with pulses of the sex pheromone [12, 21]. Patch-clamp experiments revealed that several types of Na^+^, Ca^2+^, and K^+^ ionic currents are expressed in PNs [22–25] suggesting that they may play roles in shaping the activity patterns of PNs. Especially, it was recently found that SK channels are expressed in AL PNs both in *Drosophila* [24] and in *Agrotis* [25].

A number of biophysical models of neuron and network have been developed to investigate the cellular, synaptic, network structure and dynamical mechanisms underlying PN firing patterns and odor coding in the MGC or the AL of insects. First, a simplified Hodgkin—Huxley (HH) type neuron model and a neural network model with disinhibition mechanism were developed to simulate the low-frequency (< 10 Hz) background activity and the high-frequency (> 100 Hz) bursting capacity of pheromone-sensitive PNs in moth MGC [26–27]. In the absence of stimulation, the modeled PN is inhibited by a LN (denoted LN2), the model exhibits about 3Hz spontaneous oscillations. During odor stimulation, LN2 is inhibited by another LN (denoted LN1), the PN is released from inhibition and exhibits a burst response at frequency higher than 100 Hz. *I*_Ca_ and *I*_K(Ca)_ are responsible for burst and quiescent period generation whereas *I*_A_ reduces the firing frequency. However, in this model the activation and inactivation of the ionic channels of *I*_Na_, *I*_K_, *I*_A_, *I*_Ca_ and *I*_K(Ca)_ are simplified and not biophysically realistic. Second, to simulate the temporal activity patterns induced by odor stimuli in the locust AL, neural networks with randomly connected neurons based on HH type models of PNs and LNs were developed [28–29]. In these models, *I*_Ca_ and *I*_K(Ca)_ for slow patterning generation were in non-spiking LNs, but not in PNs. The temporal patterns of PNs were generated through strong GABA_B_-mediated slow inhibition. Third, in another small neural network model of AL consisting of identical PNs and LNs of HH type, the *I*_Na_, *I*_K_, *I*_Ca_ and *I*_K(Ca)_ were located in both PNs and LNs [30]. It was found that *I*_Ca_ and *I*_K(Ca)_ in PNs are sufficient to account for the slow patterning. The authors showed that the major effect of network inhibition is to redistribute the action potentials of the PNs from bursting to one action potential per cycle of oscillation. Fourth, based on morphological and physiological data from glomerular circuitry of insect AL and using models of PNs and LNs developed in [28–29], a cross-scale neurodynamical model of the AL was developed [31]. This model demonstrates the effects of connectivity and complex dynamics in amplifying weak odor signals, in discriminating signals, and in detecting odor similarity, difference and specialty. Simulation results also showed that the spatiotemporal patterns of the odor information emerging in the glomeruli of the AL rely on the glomerular morphology, the connectivity and the complex dynamics of the AL circuits. Fifth, a model of the MGC in the moth *Manduca sexta* with HH type neuronal models and two types of inhibitory LNs, LNs-IIa, and LNs-IIb was proposed [32]. It was shown that synaptic inhibition, intrinsic currents *I*_A_ and *I*_SK_ in PNs can account for the first and second inhibitory phases and contribute to a rapid encoding of pheromone information. Sixth, recently, using a model of AL with PNs and LNs developed in [28–29], the relationship between a structural property of a network—its colorings, Ca^2+^ dynamics and the spatiotemporal activity and synchronization properties of PNs were explored [33]. Seventh, an inhibitory neural network model of MGC, which was quantitatively reduced from a HH conductance-based model to a mean field one, was recently developed [34]. It was analytically shown that the network's ability to operate on signal amplitudes across several orders of magnitude is optimal when a disinhibitory model is close to losing stability and the network dynamics are close to bifurcation.

However, most of the previous modeling work oversimplified the ORN inputs as an input current to PN. Moreover the ionic currents and parameters of the PN and LN models were not taken from insect neurons. In this work, the ORN was modeled from the experimental data recorded in the noctuid moth *A*. *ipsilon* from our lab [12]. In light of the availability of patch-clamp data of some ionic currents in PNs or other types of insect neurons [22, 36–38], we developed a biophysical model of PN. In a recent work, using a similar PN model with SK currents we reproduced the E_1_ phase and I phase [21]. In the present paper, we describe the PN model and its parameters in detail and we consider a realistic ORN input modeled from experimental data. Based on the convergence rate in the moth pheromone system [35], we connected 100 ORNs to 1 PN by fast nicotinic cholinergic synapses to form a simple model of the MGC. Our model was built based on three types of experimental data: intracellular, extracellular and patch-clamp data recorded from ORNs, PNs and other neurons in insects obtained from our lab and other labs. Because ORNs do not show triphasic patterns we simply modeled each ORN firing by an inhomogeneous Poisson process. The firing frequency of the ORN model is a function of time and is fitted to the extracellular recorded data in response to pheromone stimulations varying in concentration and duration [12]. In order to better understand the cellular and synaptic mechanisms underlying the triphasic response patterns of PNs, we made a biophysical PN model taking into account the nicotinic cholinergic currents resulting from ORN synapses onto PNs and various intrinsic ionic currents found in PNs. The parameters in the voltage-dependent steady state and time-dependent functions were fitted to patch-clamp data [22, 36]. When no data were available on PN currents we utilized data from other neuron types in insects [37–38] or even vertebrates [39]. We hypothesize that the multiphasic firing patterns of PNs may be generated by the ionic currents in PNs and ORN inputs, the cholinergic synaptic currents from ORNs to PN may affect the PN response characteristics. Using this model we reproduced the recorded triphasic response patterns of PNs. Then, we investigated the ionic current mechanisms underlying these patterns. We further performed thorough analysis on how the response characteristics change with stimulation parameters and how the ORN inputs, the intrinsic and synaptic currents affect the response characteristics. In addition, we also reconstructed a model of LN and explored possible influences of LNs on the PN response characteristics through GABA_A_- and GABA_B_-mediated inhibition. Finally, we draw some conclusions based on our modeling study.

## Results

### Reproducing the triphasic pattern and frequency of PN responses

**Fig 1:**
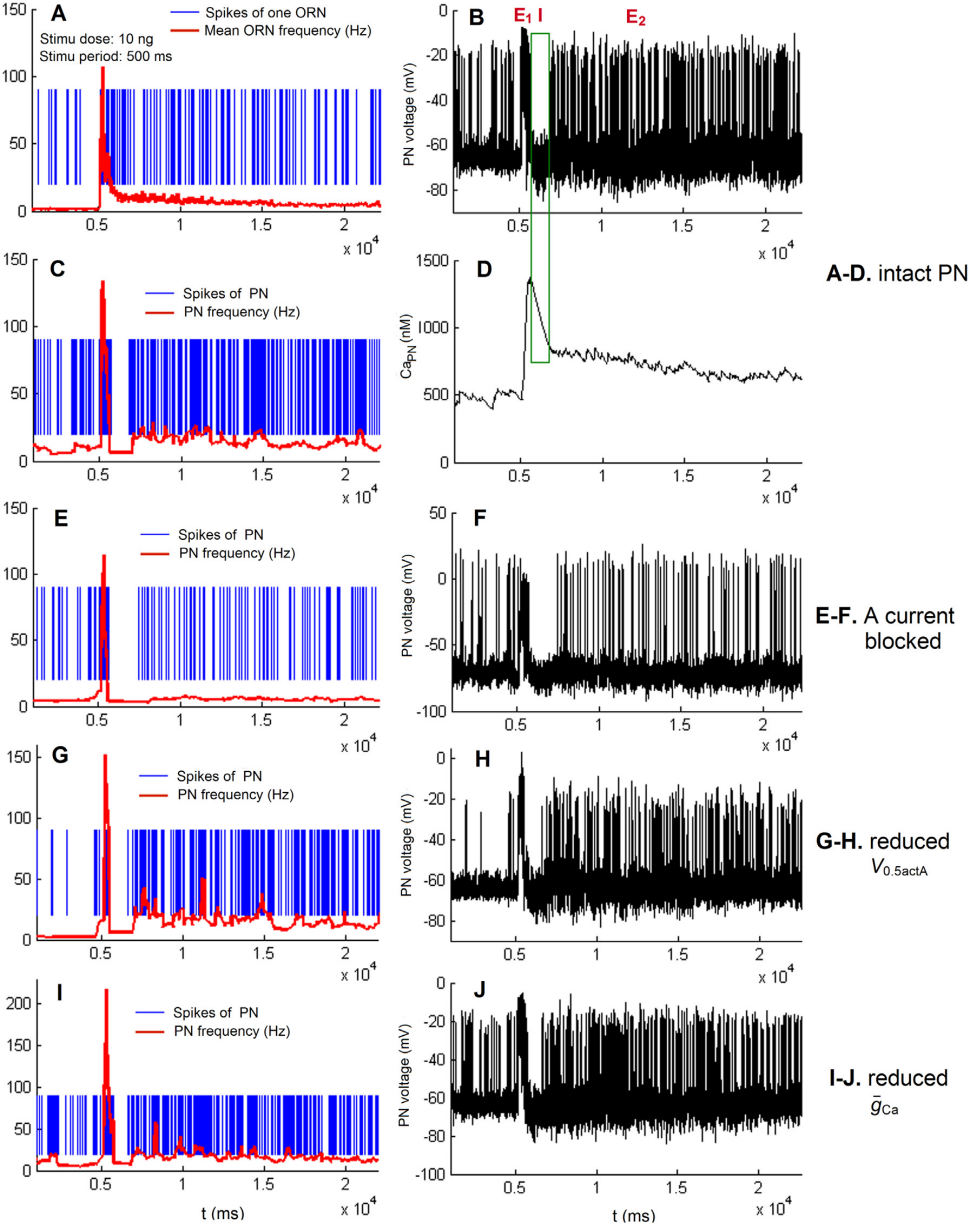
Triphasic response pattern reproduced by the MGC model in response to a pheromone stimulus (500 ms, 10ng). In the results shown in A to D, for most parameters in Eqs (4–14), we used the original values from literature given in Tables 2–4 except for some values that were modified (modified values in Tables 2–4). A. Spikes of one ORN (blue lines) and mean firing frequency curve of ORNs (red line). B. Dynamics of the PN membrane potential *V*. C. Spikes of the PN (blue lines) and PN firing frequency (red line). D. Kinetics of intracellular Ca^2+^ concentration of the PN. E. Spikes of the PN (blue lines) and PN firing frequency (red line) ($\overset{-}{g}$_A_ = 0 μS, $\overset{-}{g}$_Ca_ = 0.045 μS). F. Dynamics of the PN membrane potential *V* ($\overset{-}{g}$_A_ = 0 μS, $\overset{-}{g}$_Ca_ = 0.045 μS). G. Spikes of the PN (blue lines) and PN firing frequency (red line) ($\overset{-}{g}$_A_ = 0.5 μS, *V*_0.5actA_ = -40.0 mV, $\overset{-}{g}$_Ca_ = 0.045 μS). H. Dynamics of the PN membrane potential *V* ($\overset{-}{g}$_A_ = 0.5 μS, *V*_0.5actA_ = -40.0 mV, $\overset{-}{g}$_Ca_ = 0.045 μS). I. Spikes of the PN (blue lines) and PN firing frequency (red line) ($\overset{-}{g}$_A_ = 0.5 μS, *V*_0.5actA_ = -37.0 mV, $\overset{-}{g}$_Ca_ = 0.035 μS). J. Dynamics of the PN membrane potential *V* ($\overset{-}{g}$_A_ = 0.5 μS, *V*_0.5actA_ = -37.0 mV, $\overset{-}{g}$_Ca_ = 0.035 μS).

To understand the cellular mechanisms underlying the triphasic firing patterns (E_1_/I/E_2_) of MGC neurons in *A*. *ipsilon* in response to pheromone stimuli, we developed a simple biophysical MGC network model. This model (see Methods) consists of 100 Poisson ORNs connected to one PN through cholinergic synapses. The outline of the model is shown in S1 Fig and its parameter values are given in Tables 1–4. Using this model we reproduced the triphasic PN response pattern to high concentration pheromone stimuli. Results are shown in Fig 1A–1D. In the simulations, the stimulation onset is 5000ms and the ORN response latency is 140ms. The pheromone stimulus duration and dose are 500ms and 10ng respectively. Since the parameter values of various intrinsic currents were taken from different types of neurons, in order to produce the firing pattern shown in Fig 1A–1D we have modified the values of some parameters from the experimental data. The modified values are also shown in Tables 2–4 (denoted by modified value). Comparing Fig 1A and 1C shows that the spontaneous frequency of PN is higher than that of ORN; the E_1_ phase in PN corresponds to the initial response of ORNs where their firing frequency is the highest; the E_2_ phase in PN corresponds to the late response of ORNs where their firing rate is lower. Since the PN receives convergent inputs from 100 ORNs, the frequency of the spontaneous activity and those of E_1_ and E_2_ phases are higher in PN than in ORN. These results agree with the experimental findings in [12–13]. Fig 1B and 1D indicate that the I phase corresponds to the falling phase of intracellular Ca^2+^ of PN (shown by the green rectangle in Fig 1B and 1D). In order to see how the intrinsic current *I*_A_ affects the PN firing pattern in Fig 1E and 1F, we turned off *I*_A_. The frequency of the PN spontaneous activity and that of the second excitatory phase E_2_ in Fig 1E and 1F are reduced compared with Fig 1B and 1C. By contrast, in Fig 1G and 1H we reduced the half-activation voltage *V*_0.5act_ of *I*_A_ from -32.7 to -40.0 mV. The frequency of the second excitatory phase E_2_ was significantly increased, whereas the PN spontaneous activity was further reduced. This means that the A current affects the PN firing frequency of various phases in a parameter-dependent way. We further checked the influence of *I*_Ca_ on the firing pattern. We found that decreasing ${\overset{-}{g}}_{Ca}$, on the one hand, enhances the firing frequency during spontaneous activity, as well as the E_1_ and E_2_ phases; on the other hand, it decreases the duration of the I phase. One of the results is shown in Fig 1I and 1J. In the subsection “Effects of intrinsic PN parameters on the response characteristics”, the influence of PN intrinsic currents is detailed.

**Table 1:** Parameter values of ORN model fitted to extracellular recorded data.

Dose (ng)	Period (ms)	*f*_sp_ (Hz)	*f*_pe_ (Hz)	*f*_*pl*_ (Hz)	*T*_lat_ (ms)	*T*_d2pe_ (ms)	*T*_pl_ (ms)	τ_rise_ (ms)	τ_fall1_ (ms)	τ_fall2_ (s)	τ_fall3_ (s)	*q*
0.1	200	1.5	16	—	250	150	—	180	130	20	—	0.9
1.0	200	1.5	35	—	250	115	—	128.6	170	10	—	0.9
10.0	200	1.5	154	—	150	115	—	155	115	5	—	0.9
10.0	500	1.5	125	30	140	160	330	150	40	0.2	10.5	0.72
10.0	1000	1.5	130	30	170	110	870	140	70	0.3	11.791	0.72

**Table 2:** Parameter values of *C*a dynamics and passive parameters of the PN model.

	Name	Value	Modified value	Reference
Passive parameters	*C*_m_(pF)	22.9	**--**	[22]
	*E*_L_ (mV)	-61.4	**--**	[22]
	*g*_*L*_ (nS)	11.16^a^	**--**	[22]
Parameters of *C*a dynamics	*f*_Ca_	1.6	1.7	[39]
	*τ*_Ca_ (ms)	656	2000	[39]
	*Ca*_∞_(nM)	113.0	**--**	[39]

^a^Calculated by *g*_L_ = 1/*R*_M_ = 1/(89.6 MΩ) = 11.16 nS.

**Table 3:** Parameter values of the ionic currents of the PN model given or calculated from data.

Steady-state functions of *I*_Na_	${\overset{-}{g}}_{Na}$(nS)	*V*_0.5act_ (mV)	*s*_m_	*V*_0.5inact_ (mV)	*s*_h_	*E*_Na_(mV)	Ref.
	206 modified2500	-25.8	9.32	-41.1 modified43.0	9.75	+48.2(RP)+47.9(EP)	[38]
Time constant functions of *I*_Na_	*a*_τm,up_	*V*_τm,0.5up (mV)_	*s*_τm,up_	*a*_τm,dn_	*V*_τm,0.5dn_	*s*_τm,dn_	Ref.
	0.5	-30	3.7	0.5	-15	13.7	Fitted
	*a*_τh,up_	*V*_τh,0.5up_	*s*_τh,up_	*a*_τh,dn_	*V*_τh,0.5dn_	*s*_τh,dn_	to
	2.1	-55	5	0.7	-10	11	[38]
Steady-state functions of *I*_Ca_	${\overset{-}{g}}_{Ca}$(nS)	*V*_0.5act_ (mV)	*s*_m_	*V*_0.5inact_ (mV)	*s*_h_	E_Ca_(mV)	Ref.
	16.1 modified45	-10.6	8.5	-29.6	8.4	160	[22]
Time constant functions of *I*_Ca_	*a*_τm,up_	*s*_τm,up_	*a*_τm,dn_	*V*_τm,dn_	*s*_τm,dn_	**—**	Ref.
	0.046	20.73	0.19	19.8	10	**—**	[39]
Steady-state functions of *I*_sk_	*a*_msk_	*b*_msk_	*s*_msk_	**—**	**—**	**—**	Ref.
	1.120	2.508	1000	**—**	**—**	**—**	[39]
Steady-state functions of *I*_Kd_	${\overset{-}{g}}_{Kd}$ (nS)	*V*_0.5act_ (mV)	*s*_m_	E_K_ (mV)	**—**	**—**	Ref.
	8.17^a^ modified700	-18.5	22.5 modified 20.0	-91.6	**—**	**—**	[36]
Time constant functions of *I*_Kd_	*a*_τm,up_	*V*_τm,0.5up_	*s*_τm,up_	**—**	**—**	**—**	Ref.
	0.125	-40	11.0	**—**	**—**	**—**	Fitted
	*a*_τm,dn_	*V*_τm,0.5dn_	*s*_τm,dn_	**—**	**—**	**—**	to
	0.15	25	45.7	**—**	**—**	**—**	[37]
Steady-state functions of *I*_A_	${\overset{-}{g}}_{A}$(nS)	*V*_0.5act_ (mV)	*s*_m_	*V*_0.5inact_ (mV)	*s*_h_	E_K_(mV)	Ref.
	17.35^b^ modified 500	-32.69	17.5	-53.3	7.23	-91.6	[36]
Time constant functions of *I*_A_	*a*_τm,up_	*V*_τm,0.5up_	*s*_τm,up_	*a*_τm,dn_	*V*_τm,0.5dn_	*s*_τm,dn_	Ref.
	0.5	-30	13.7	0.42	-15	46	Fitted
	*a*_τh,up_	*V*_τh,0.5up_	*s*_τh,up_	*a*_τh,dn_	*V*_τh,0.5dn_	*s*_τh,dn_	to
	0.04	-55	25	0.045	40	55	[37]

^a,b^Calculated from Kloppenburg et al.,1999.

**Table 4:** Parameter values of nACh synaptic current.

Name	$\overset{-}{g}$_nACh_(μS)	*E*_nACh_ (mV)	*α* (ms^-1^)	*β* (ms^-1^)	*A*	*t*_max_ (ms)
Value	0.3	0.0	10	0.2	0.5	0.3
Reference	[28]	[28]	[28]	[28]	[28]	[28]
Modified value	0.017	--	--	2.0	0.8	--

**Fig 2:**
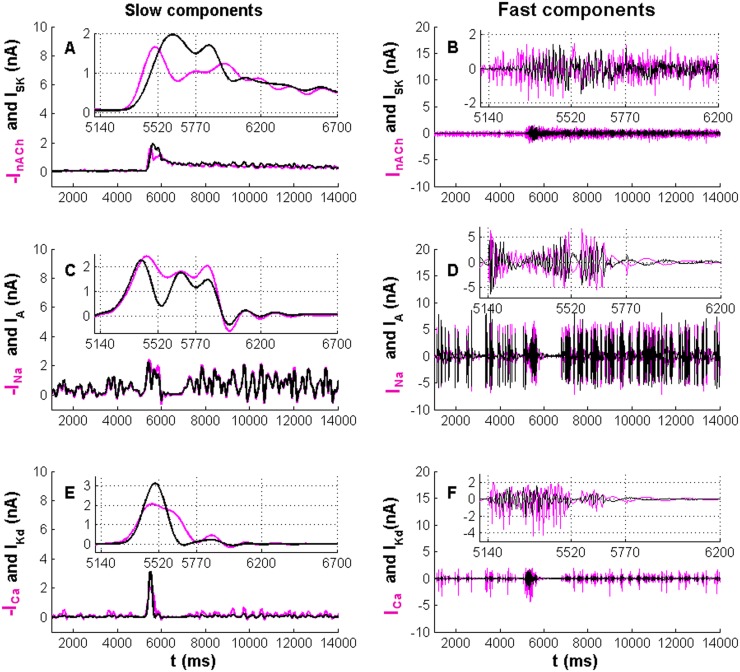
Synaptic and intrinsic currents in a PN from the simulation results shown in Fig 1. Left panel: the slow components of the repolarizing currents (black lines) and depolarizing currents (magenta lines); *I*_SK_ and-*I*_nACh_ (A), *I*_A_ and—*I*_Na_ (C) and *I*_Kd_ and—*I*_Ca_ (E). Right panel: Fast components of the repolarizing (black lines) and depolarizing currents (magenta lines); *I*_SK_ and *I*_nACh_ (B), *I*_A_ and *I*_Na_ (D) and *I*_Kd_ and *I*_Ca_ (F). Note that in the left panel we draw the minus values of *I*_nACh_, *I*_Na_, *I*_Ca_ for comparing their amplitudes, while in the right panel we draw the values of *I*_nACh_, *I*_Na_, *I*_Ca_ directly for comparing their depolarizing and repolarizing effects). The slow components of E_1_ (from 5140 to 5770ms) and I (from 5770 to 6700ms) are enlarged in the insets in A, C and E; and the fast components of E_1_ and the period transiting from E_1_ to I (from 5770 to 6200ms) are enlarged in the insets in B, D and F.

In order to further reveal the mechanisms underlying the generation of the E_1_/I/E_2_ pattern, we analyzed the PN depolarizing and repolarizing currents in our simulation (Fig 1A–1D). Employing the low-pass (cut-off frequency: 5Hz) and high-pass (cut-off frequency: 5Hz) Butterworth filters (see Methods), we extracted the slow and fast components of the depolarizing currents *I*_nACh_, *I*_Na_, *I*_Ca_ and the repolarizing currents *I*_SK_, *I*_A_ and *I*_Kd_ (Fig 2). During E_1_ and I, the kinetics of the slow component of the repolarizing Ca^2+^-dependent K^+^ current *I*_SK_ are similar to those of the depolarizing synaptic current *I*_nACh_ (Fig 2A). Similarly, during E_1_, the kinetics of the slow components of *I*_A_ and *I*_Kd_ are similar to those of the *I*_Na_ and *I*_Ca_, respectively (Fig 2C and 2E). In the beginning of E_1_ (from 5140 to 5520ms) the amplitudes of the slow and fast components of the depolarizing synaptic current *I*_nACh_ from ORNs are higher than those of the repolarizing current *I*_SK_ (insets in Fig 2A and 2B). With depolarization *I*_SK_ increases because of the accumulation of intracellular Ca^2+^. At 5520ms the amplitude of the slow component of *I*_SK_ exceeds that of *I*_nACh_ (inset in Fig 2A). From 5520ms onwards *I*_SK_ competes with *I*_nACh_ and slows down the PN firing. At 5770ms the PN stops spiking and transits to the I phase. During I, *I*_SK_ flows out against *I*_nACh_ to create a hyperpolarization phase until the intracellular Ca^2+^ concentration falls. During this hyperpolarization phase, the voltage-gated currents (*I*_A_, *I*_Kd_, *I*_Na_ and *I*_Ca_) cannot be activated due to the low membrane potential (Fig 2C–2F). Interestingly, both the slow and fast components of *I*_A_ have the same kinetics as *I*_Na_ and the amplitude of *I*_A_ is slightly smaller than that of *I*_Na_, especially during E_1_ and E_2_ phases (Fig 2C and 2D and the insets). Whenever *I*_Na_ depolarizes the membrane to make a spike, *I*_A_ flows out and repolarizes the membrane, so that the amplitude of each spike is reduced and the firing frequency is increased.

### Effects of the pheromone stimulus parameters on the response characteristics

**Fig 3:**
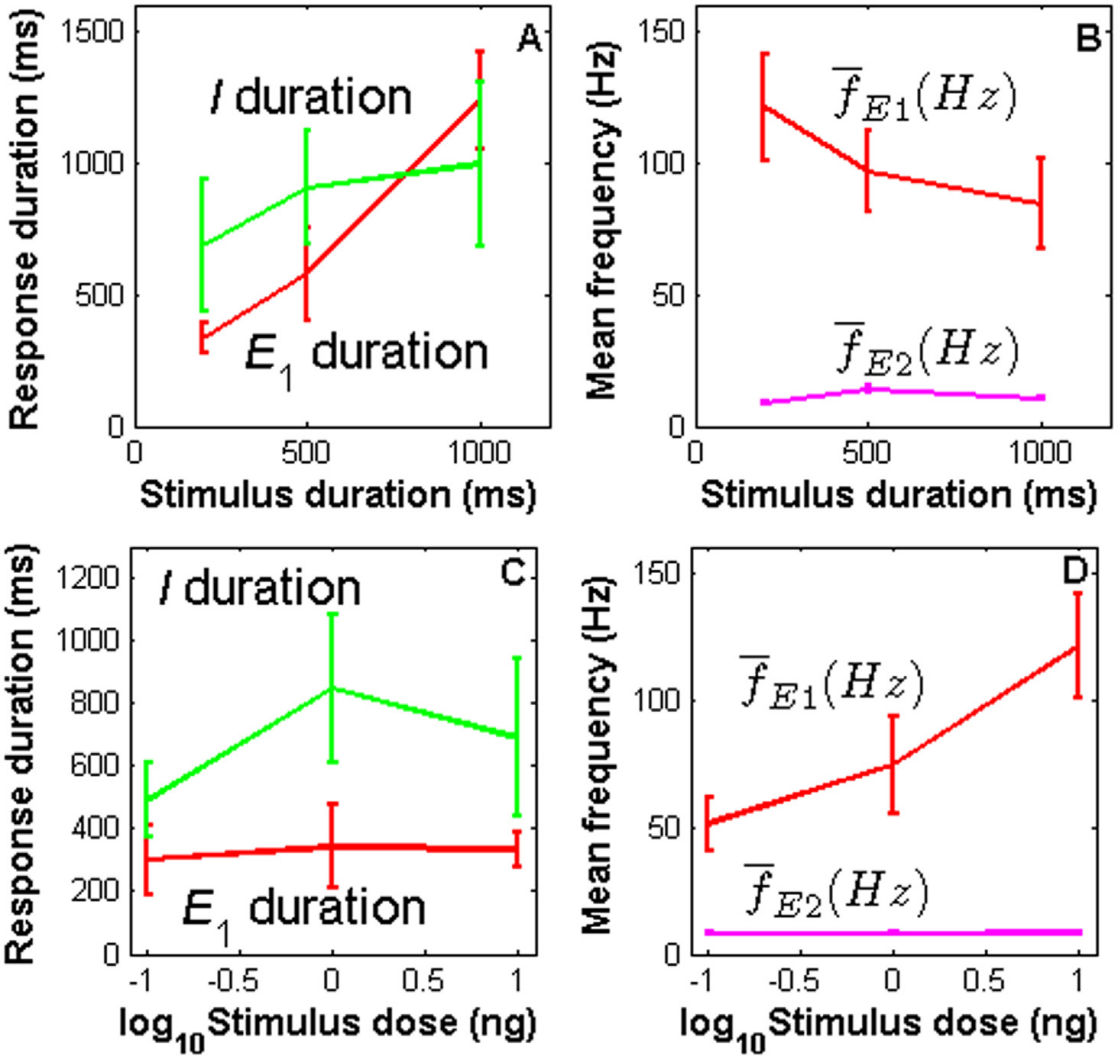
Effects of stimulation parameters on PN response characteristics. Top panel: effects of stimulus duration on E_1_ and I duration (A) and mean firing frequency of E_1_ and E_2_ (B). Bottom panel: effects of stimulus concentration on E_1_ and I durations (C) and mean firing frequency of E_1_ and E_2_ phases (D).

To see how the stimulation parameters affect the PN response characteristics (such as duration and frequency) whose calculation is described in Methods section, we varied the concentration and duration of the pheromone pulses. For each parameter value, the computer simulation was repeated ten times and the mean and standard deviation of the response characteristics were calculated over the ten trials. The results are shown in Fig 3. The parameter values are the same as those in Fig 1A–1D). The duration of E_1_ nearly linearly increases with the stimulus duration and the duration of I for 200ms stimulus duration is slightly lower than that for 500 and 1000ms stimulus duration (Fig 3A). The mean response frequency of E_1_ decreases with stimulus duration (this is due to frequency adaptation during E_1_) (Fig 3B). E_1_ and I durations are independent of stimulation doses (Fig 3C). These relationships between E_1_ duration and stimulation duration and dose agree with the experimental findings described in [12]. The mean response frequency of E_1_ increases with stimulus concentration while that of E_2_ does not (Fig 3D).

### Effects of intrinsic PN parameters on the response characteristics

We examined how the intracellular Ca^2+^ dynamics and intrinsic currents in the PN affect its dynamic response characteristics. To this end we varied the value of each parameter from low to high in a range while keeping the values of other parameters the same as in Fig 1A–1D. At a given value of each parameter the computer simulation was repeated ten times and the mean and standard deviation of each response characteristics were calculated over the ten trials.

#### Effects of Ca^2+^ dynamics, I_Ca_ and I_SK_

**Fig 4:**
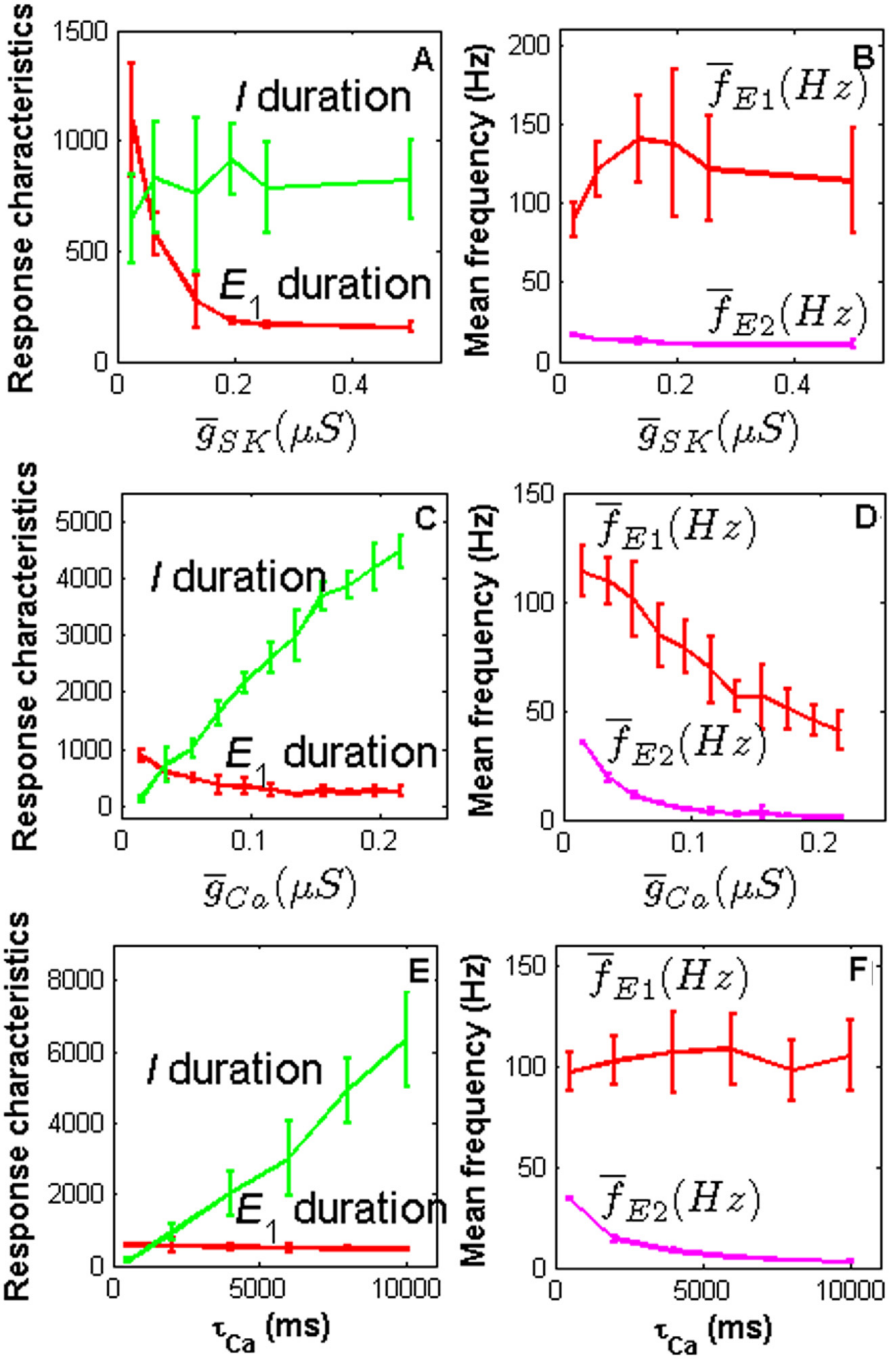
Effects of *I*_Ca_, *I*_SK_ and dynamics of intracellular Ca^2+^ on PN response characteristics. Top panel: effects of the mean maximal conductance $\overset{-}{g}$_SK_ of *I*_SK_ on E_1_ and I durations (A) and mean firing frequency of E_1_ and E_2_ (B). Intermediate panel: effects of the mean maximal conductance $\overset{-}{g}$_Ca_ of *I*_Ca_ on E_1_ and I duration (C) and mean firing frequency of E_1_ and E_2_ (D). Bottom panel: effects of time constant τ_*Ca*_ of Ca^2+^ dynamics on E_1_ and I duration (E) and mean firing frequency of E_1_ and E_2_ phases (F).

The simulation results indicate that the inhibitory phase following E_1_ is generated by the slow component of the repolarizing current *I*_SK_ that exceeds the slow component of the depolarizing synaptic current *I*_nACh_. *I*_SK_ depends on Ca^2+^ concentration which in turn depends on *I*_Ca_. In addition, *I*_Ca_ also affects the triphasic response pattern (Fig 1I–1J). Therefore we further analyzed the effects of the parameters of the Ca^2+^ dynamics, *I*_Ca_ and *I*_SK_ on the PN response characteristics. Fig 4 shows that the duration of the E_1_ phase exponentially decreases with $\overset{-}{g}$_SK_ (A) while the duration of the I phase linearly increases with $\overset{-}{g}$_Ca_ (C) and τ_Ca_ (E). The mean frequency of the E_1_ phase linearly decreases with $\overset{-}{g}$_Ca_ (D) and that of the E_2_ phase exponentially decreases with $\overset{-}{g}$_Ca_ (D) and τ_*Ca*_ (F). Some parameters for the steady-state activation *m*_∞_, the time constant τ_m_ of *I*_Ca_ and for the steady-state function of the gating variable m_sk∞_ of *I*_SK_ have also clear influences on the PN response duration, especially the duration of the I phase. These effects are illustrated in S2 Fig. The I duration decreases with *a*_τm,up_ and *S*_msk_, while it increases with *S*_τm,up_ and *a*_msk_.

#### Effects of INa, IA and Ikd

**Fig 5:**
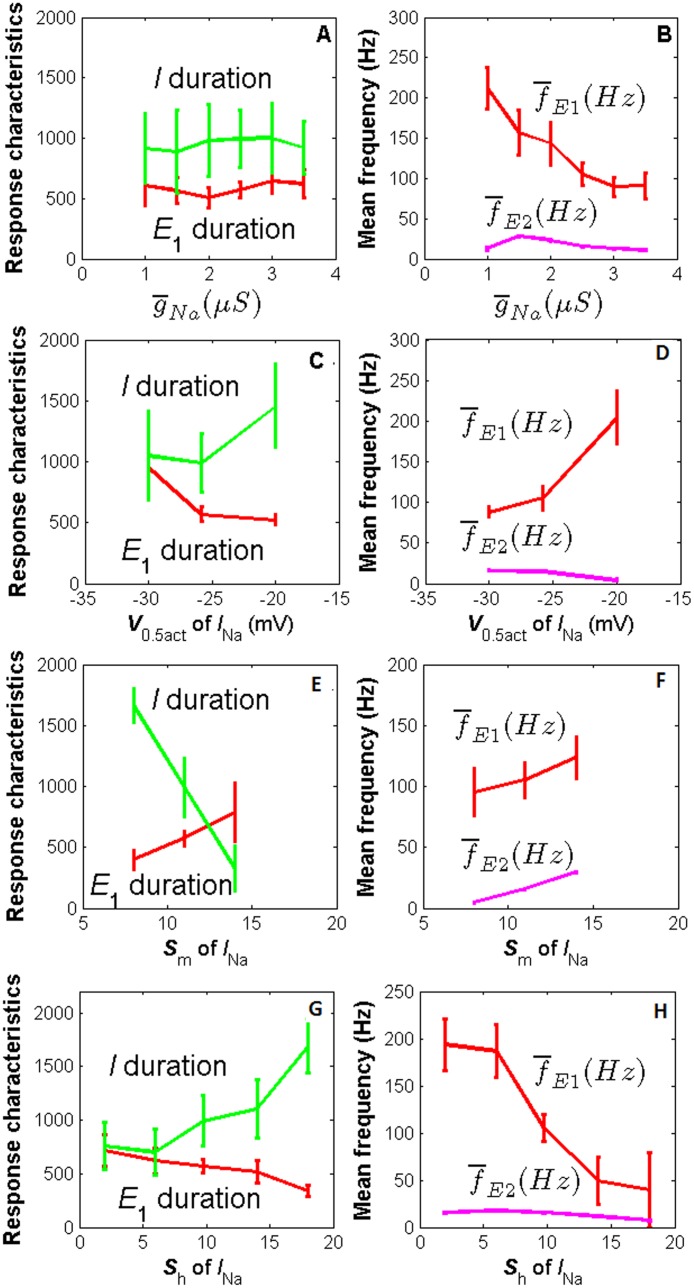
Major effects of *I*_Na_ on PN response characteristics. Effects of $\overset{-}{g}$_Na_, *V*_0.5act_, *S*_m_ and *S*_h_ on E_1_ and I duration (left panel) and mean firing frequency of E_1_ and E_2_ (right panel).

**Fig 6:**
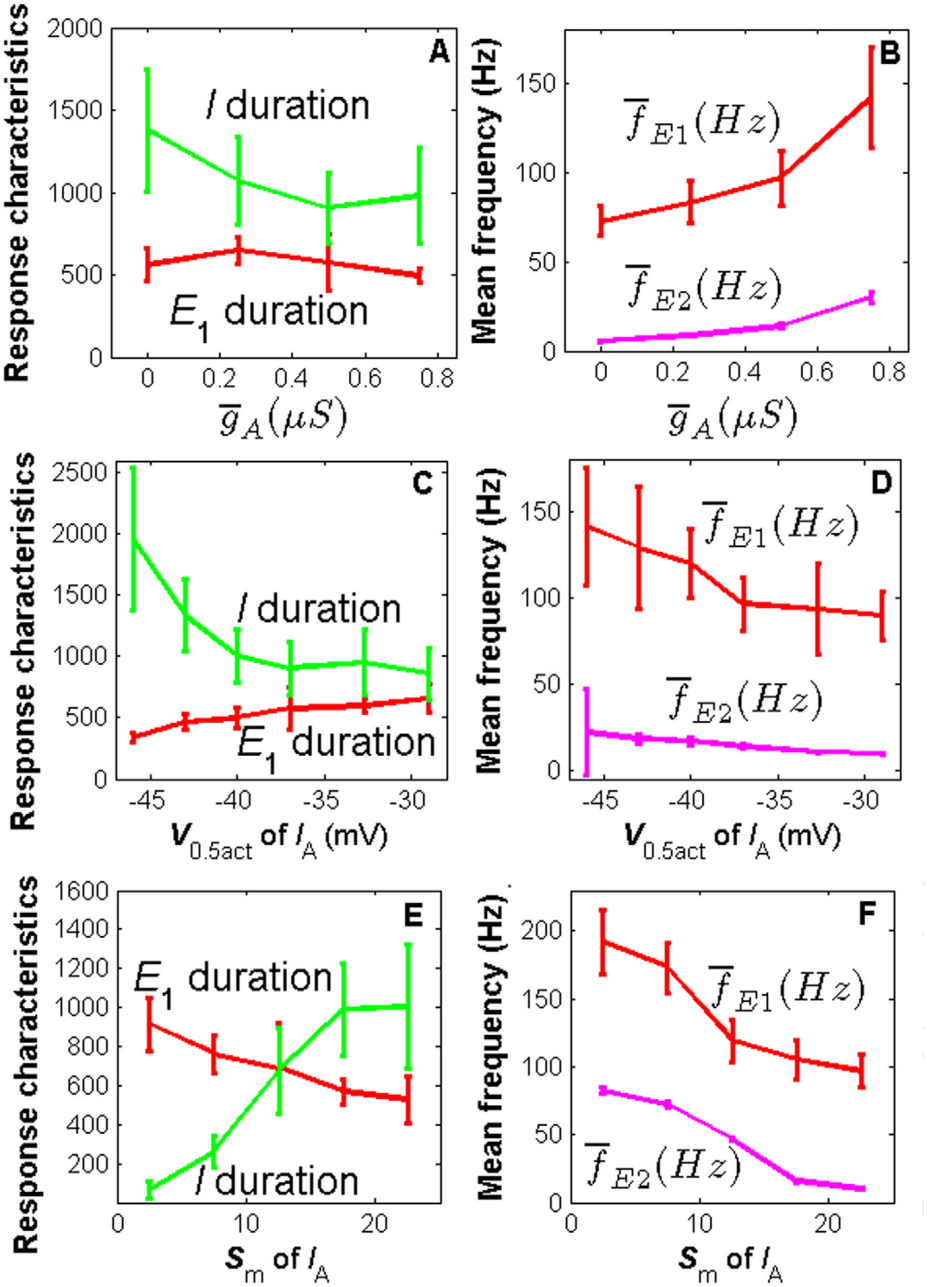
Major effects of *I*_A_ on PN response characteristics. Top panel: effects of $\overset{-}{g}$_A_ on E_1_ and I duration (A) and mean firing frequency of E_1_ and E_2_ (B). Intermediate panel: effects of *V*_0.5act_ on E_1_ and I duration (C) and mean firing frequency of E_1_ and E_2_ phases (D). Bottom panel: effects of *S*_m_ on E_1_ and I duration (E) and mean firing frequency of E_1_ and E_2_ phases (F).

In this section, by varying the parameter values of *I*_Na_, *I*_*kd*_ and *I*_A_, we quantitatively investigated how the PN response characteristics depend on these parameters. The major influences of *I*_Na_ on the response characteristics are illustrated in Fig 5. The maximal conductance and parameters for steady-state activation and inactivation function of *I*_Na_ strongly affect the response frequency of E_1_ phase: E_1_ frequency increases with the half-activation parameter *V*_0.5act_ (Fig 5D) and decreases with $\overset{-}{g}$_Na_ (Fig 5B) and the slope factor *S*_h_ (Fig 5H); both E_1_ and E_2_ frequencies increase with the slope factor *S*_m_ (Fig 5F). Parameters *V*_0.5act_, *S*_m_ and *S*_h_ of *I*_Na_ have also some influences on E_1_ and I duration (Fig 5C, 5E and 5G). Other parameters of *I*_Na_ affect one or two PN response characteristics as shown in S3 Fig. *I*_Kd_ has also some influences on the PN response characteristics as shown in S4 Fig. As illustrated in Fig 1 the transient potassium current *I*_A_ affects the firing frequency of E_1_, E_2_. Here we further investigated the influences of *I*_A_. We found that the mean maximal conductance, the half activation, and the slope factor *S*_m_ of *I*_A_ have strong effects on PN response frequency. Increasing the maximal mean conductance $\overset{-}{g}$_A_ and decreasing the voltage of half activation*V*_0.5act_ and the slope factor *S*_m_ increase the mean firing frequency of both E_1_ and E_2_ phases (Fig 6B, 6D and 6F). The increased frequency of spontaneous activity of the PN is also due to the higher conductance and lower voltage of half activation and smaller slope factor *s*_m_ of this current (data not shown). In addition, increasing $\overset{-}{g}$_A_ has a small effect of decreasing the duration of the I phase at low $\;\overset{-}{g}$_A_ values (Fig 6A); increasing *V*_0.5act_ clearly decreases the duration of the I phase and increases the duration of the E_1_ phase (Fig 6C). Moreover, decreasing *S*_m_ clearly decreases I duration whereas increases E_1_ duation. Some parameters in time constant functions of *I*_A_ have slight influences on PN reponse characteristics (data not shown): I duration increases with *a*_*τm*_,_up_, *a*_*τh*_,_dn_ and *V*_*τm*_,_0.5dn_ when it is less than -10 mV while decreases with *S*_*τm*_,_up_, *a*_*τh*_,_up_ and *V*_*τh*_,_0.5dn_ when it is less than -40 mV; E_1_ duration decreases with *a*_*τm*_,_up_ and E_1_ frequency decreases with *V*_*τm*_,_0.5up_, *S*_*τm*_,_dn_, *a*_*τh*_,_up_, *S*_*τh*_,_up_ and *a*_*τh*_,_dn_; E_2_ frequency increases with *S*_*τm*_,_up_ and *a*_*τm*_,_dn_ when it is less than 0.7.

### Effect of nACh synaptic parameters on PN firing patterns

In the presence or absence of pheromone stimulation, PN dendrites receive feedforward cholinergic synaptic inputs from ORNs through nicotinic receptors. Hence, the nACh synaptic currents are the stimulation inputs of PNs. We studied how the parameters of *I*_nACh_ affect the PN response characteristics.

First, the effects of presynaptic ACh transmitter delivered as square pulses of duration *t*_max_ and concentration *A* were investigated (S5 Fig). E_1_ duration increases while I duration decreases with Ach pulse duration *t*_max_ (S5A Fig). The mean frequency of the E_2_ phase ${\overset{-}{f}}_{E2}\;$ significantly increases with *t*_max_, whereas the influence of this parameter on the average frequency of the E_1_ phase ${\overset{-}{f}}_{E1}$ is not monotonic (S5B Fig). This result is interesting because this parameter has a different effect on the average frequency of the E_1_ and E_2_ phases. The ${\overset{-}{f}}_{E1}$ increases at values of *t*_max_ smaller than 0.3ms then decreases with *t*_max_. This is due to the fact that the firing frequency adaptation induced by SK currents takes effect when the E_1_ duration increases with *t*_max_. The ACh concentration parameter *A* has a very different effect on the duration of E_1_ and I phase and the average frequency of E_1_ phase ${\overset{-}{f}}_{E1}$. The duration of E_1_ phase increases and that of I phase decreases with *A* when *A* is less than 1, then the duration of E_1_ and I phases reaches saturation (S5C Fig). The clearest effect of *A* is that it can significantly increase the average frequency of E_1_ phase ${\overset{-}{f}}_{E1}$ (S5D Fig).

Second, we investigated the effects of the opening and closing rates of nACh postsynaptic channels on PN response characteristics (S6A–S6D Fig). Increasing the opening rate α slightly increases E_1_ duration and decreases I duration (S6A Fig) and significantly increases the mean frequency of E_2_ phase (S6B Fig purple line). Increasing the closing rate β has exactly opposite effects (S6C and S6D Fig purple line). Interestingly, increasing α and β has the same clear effect of increasing the average frequency of the E_1_ phase (S6B and S6D Fig red lines). The increasing effect of β on ${\overset{-}{f}}_{E1}$ can be explained by its decreasing effect on E_1_ duration. When the value of β is small, the E_1_ duration is quite long (S6C Fig). Thus the frequency adaptation mediated by SK currents is strong. As a result of frequency adaptation, the mean frequency of E_1_ phase is low.

Finally, the effects of the mean maximal conductance $\overset{-}{g}$_nACh_ on PN response characteristics were studied. Increasing $\overset{-}{g}$_nACh_ clearly increases the duration of the E_1_ phase and strongly decreases that of the I phase (S6E Fig) and significantly increases the mean frequency of both phases (S6F Fig).

### Exploring possible effects of LNs on PN response patterns

In order to investigate possible network mechanisms, particularly the influences from LNs we have developed a model of type I LNs (LNIs), that generate Na^+^-driven action potentials [22–23]. The mathematical description of the LNI model and its parameter values are given in S1 Text. We have done some exploratory simulations by connecting 80 ORNs and 20 LNI with one PN. The LNIs receive inputs from ORNs and PN through fast nicotinic cholinergic synapses, while the PN receives fast nicotinic cholinergic synaptic inputs from ORNs and fast GABAergic inhibitory inputs from LNIs mediated by GABA_A_ receptors or slow GABAergic inhibitory inputs from LNIs mediated by metabotropic GABA_B_ receptors. Preliminary results revealed that the synaptic interactions between PN and LNIs affect the synchronization among the PN and LNIs, PN response pattern and PN response characteristics. Synchronization and I duration changes with fast GABAergic inhibition. S7 Fig qualitatively shows how the closing rate β of the GABA_A_ synaptic currents from LNI to PN affects the response characteristics and synchronization. Decreasing β decreases the synchronization of PN and LNIs and also decreases the duration of I phase. As for the influence of slow GABAergic inhibition, we found that in the parameter range of the slow GABAergic inhibition given in S1 Text (Eq S3) the PN maintained the triphasic response pattern (S8A Fig). Decreasing the rate parameter *r*_3_ in Eq S3 prolonged the I duration (S8E Fig). In these cases the rising kinetics of G protein concentration is close to that of the intracellular Ca^2+^ concentration in PN (S8C and S8F Fig). The LNIs showed synchronized triphasic response pattern (S8B and S8D Fig). When increasing *r*_3_ and decreasing *r*_4_ in Eq S3 the rising kinetics of GABA_B_ receptor-coupled G protein concentration became faster than that of the intracellular Ca^2+^ concentration in PN. In this case the E_1_ phase of PN may become an I phase (data not shown) or terminate early due to the GABAergic inhibition (S8G Fig). The shortened or disappeared E_1_ is at odds with the experimental findings in [12] and S9 Fig showing that the E_1_ duration lasts approximately as long as the stimulus and increases with the duration of pheromone stimuli. After an I phase, another excitatory phase appeared (S8G Fig) which corresponds to the rising period of intracellular Ca^2+^ concentration (S8H Fig). The second excitatory phase in turn is followed by another short I phase corresponding to the early falling phase of Ca^2+^ concentration. After the short I phase, the late excitatory phase of PN appeared.

## Discussion

In this work, using a simple MGC model with ORNs based on the recorded frequency curves under different pheromone stimuli, PN based on patch-clamp data from PN and other neurons and a biophysical model of nACh synapses, we reproduced the triphasic response pattern of PNs at high pheromone stimulation concentrations. We investigated mechanisms generating this triphasic response pattern. Our results show that it can be shaped by intrinsic mechanisms in ORNs and PNs: in our model Ca^2+^-dependent SK current in PN is responsible for the I phase following the E_1_ phase and the E_2_ phase is due to the long-lasting excitatory response of ORNs. We further investigated how the external stimulation parameters and the parameters of the internal ionic currents in PN and the nACh synaptic currents from ORNs to PN affect the duration of E_1_ and I, the firing frequency of E_1_ and E_2_ and other response characteristics of PN. In our model E_1_ duration significantly increases with stimulation duration. The ending time of E_1_ clearly depends on parameters: ${\overset{-}{g}}_{SK};\;{\overset{-}{g}}_{Ca}$; *V*_0.5act_, *S*_m_ and *S*_h_ of *I*_Na_; *t*_max_, *A*, *β* and $\overset{-}{g}$_nACh_ of *I*_nACh_. The external stimulation parameters have no significant influence on I duration. This implies that I phase is an intrinsic property of the network. Our results revealed that I duration linearly increases with the time constant of intracellular Ca^2+^ (τ_Ca_) and ${\overset{-}{g}}_{Ca}$, decreases with *t*_max_ and $\overset{-}{g}$_nACh_ of *I*_nACh_. I duration is also influenced by some other parameters such as *S*_m_, *S*_τm,up_ of *I*_Na_; a_τm,up_, *S*_τm,up_ of *I*_Ca_; *a*_msk_ and *S*_msk_ of *I*_SK_; $\overset{-}{g}$_A_ and *V*_0.5act_ of *I*_A_; and the concentration of the pulse of ACh transmitter delivered. The mean firing frequency of E_1_ phase ${\overset{-}{f}}_{E1}$ increases with stimulation concentration and decreases with stimulation duration. ${\overset{-}{f}}_{E1}$ and ${\overset{-}{f}}_{E2}$ are also strongly affected by some intrinsic parameters. Both of ${\overset{-}{f}}_{E1}$ and ${\overset{-}{f}}_{E2}$ increase with *S*_m_ of *I*_Na_. ${\overset{-}{f}}_{E1}$ increases with *V*_0.5act_ of *I*_Na_; $\overset{-}{g}$_A; $\overset{-}{g}$ nACh_, *A*, α and *β* of *I*_nACh_; while decreases with ${\overset{-}{g}}_{Na}$, *S*_h_ and *V*_0.5inact_ of *I*_Na_; *V*_0.5act_ and *S*_m_ of *I*_Kd_; ${\overset{-}{g}}_{Ca}$; and *V*_0.5act_ of *I*_A_. Besides, $\overset{-}{g}$_SK_ and *t*_max_ have non monotonic effects on ${\overset{-}{f}}_{E1}.\;{\overset{-}{f}}_{E2}$ increases with $\overset{-}{g}$_A_, *t*_max_, *A*, α and $\overset{-}{g}$_nACh_, while it decreases with ${\overset{-}{g}}_{Ca}$, τ_Ca_ and *β*.

The aim of this study is to show that it is possible to explain the triphasic PN responses with intrinsic mechanisms only (and realistic parameter values) without denying the possible implication of extrinsic mechanisms and influences. We have also explored possible network effects on the PN response characteristics, particularly the influences from LNs. Preliminary results show that I duration and synchronization between PN and LNs change with GABAergic inhibition. Slow GABAergic inhibition does not affect the triphasic PN response pattern in the parameter range given in S1 and S2 Texts. When the rising kinetics of GABA_B_ receptor-coupled G protein concentration became faster than that of the intracellular Ca^2+^ concentration in PN, GABA_B_ mediated inhibition may change the triphasic pattern and result in a much reduced E_1_ duration which is at odds with the experimental data. This might indicate that in the MGC of *A*. *ipsilon* moths if GABA_B_ receptor mediated slow inhibitory synapses exist GABA_B_ receptor may couple to Ca^2+^ activated SK channel via G protein as reviewed in [41]. Previously we have found that applying bicuculline (BIC), an antagonist of GABA_A_ and a SK channel blocker, to *A*. *ipsilon* moths abolished the inhibitory phase in all tested neurons [21]. However, applying picrotoxin (PTX), another GABA_A_ antagonist, to *A*. *ipsilon* led to different effects. This experimental result together with our modeling results indicate that the Ca^2+^-activated SK channel is likely responsible for the generation of I phase while interactions between LNs and PNs might affect the PN response characteristics. These are very preliminary qualitative results showing the influences of LNs on PN response pattern. The network structure of MGC may also affect the PN response characteristics. Further experimental and modeling investigations are needed to study how the intrinsic property of LNs, various synaptic mechanisms and network structure of MGC affect PN response. Besides, the functional roles of PN response patterns in the dynamical representation, classification and discrimination of pheromone stimuli and in guiding the moths tracking in turbulent and intermittent pheromone plumes to be elucidated.

In conclusion, our modeling study revealed that *I*_SK_ and the long-lasting excitatory response of ORNs can be intrinsic mechanisms for the generation of triphasic response patterns of pheromone-sensitive PN. The parameters of Ca^2+^, nACh synaptic, Na^+^ and A currents have strong influence on the response patterns and the response characteristics. Preliminary results show that network interactions between PN and LNIs can also affect the PN response. Although SK channels can be responsible for the generations of I phase, parameters of *I*_Ca_, *I*_Na_ and *I*_A_, as well as the synaptic currents can also affect the I phase. Therefore, experimentally blockers that affect any of these parameters might block the I phase.

## Methods

Based on various experimental findings (see S2 text, Experimental findings in ORNs and PNs), we developed models of ORN and PN and of the MGC neural network. The model parameters were fitted to the experimental data.

### The Poisson model of ORNs

**Fig 7:**
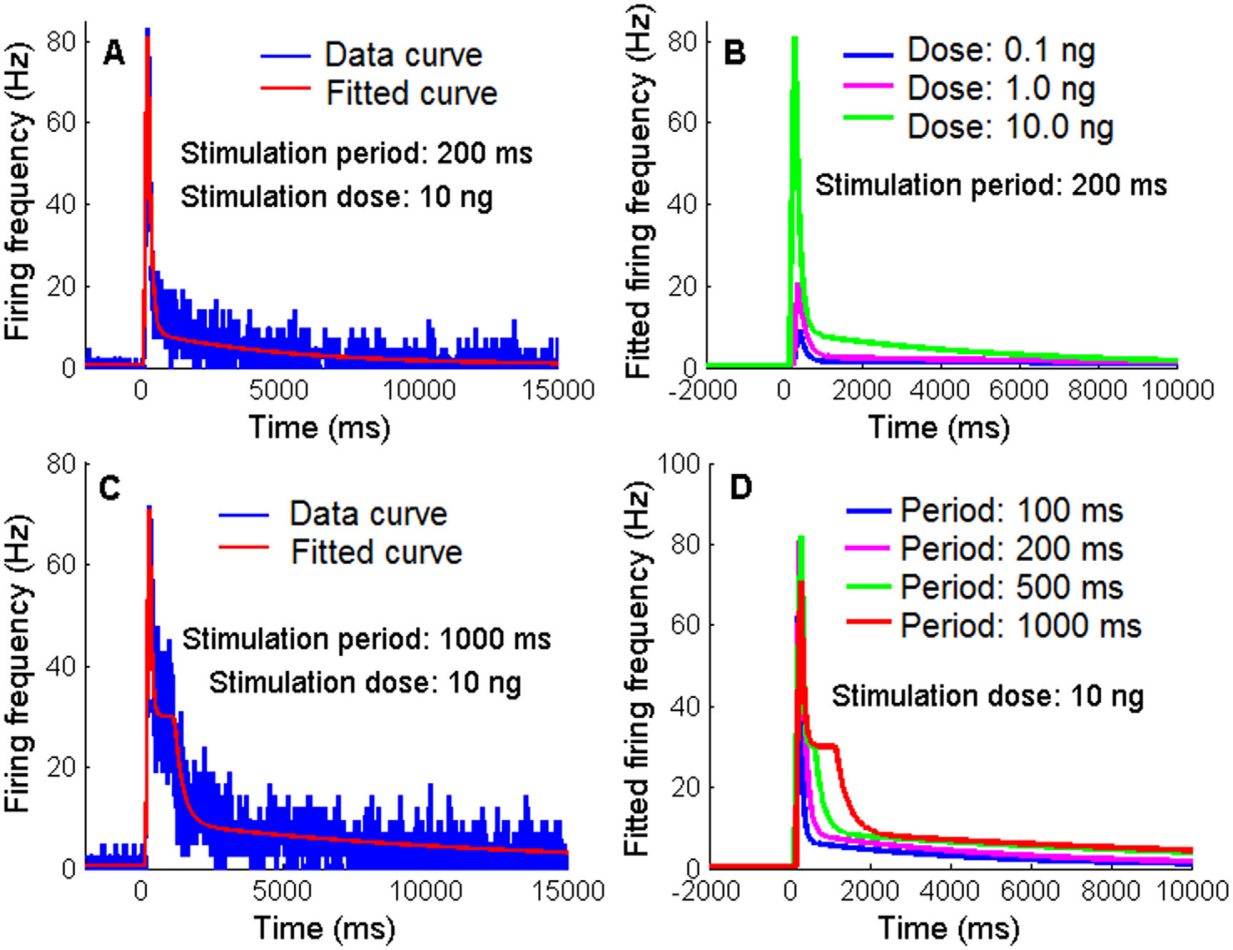
Mean frequency response curves of the ORN population in response to different concentrations and durations of the pheromone stimulation. A. Response data curve (blue) and fitted curve (red) to stimulus: 10 ng and 200 ms. B. Fitted response curves to the same stimulation period 200 ms and different stimulation doses from 0.1 to 10 ng. C. Response data curve (blue) and fitted curve (red) to stimulus: 10 ng and 1000 ms. D. Fitted response curves to the same stimulation dose 10 ng and different stimulation periods from 100 to 1000 ms.

To construct the Poisson model of ORNs, we fitted the extracellular recorded data [12] about the rise and fall of the mean instantaneous response frequency as a function of time following different concentrations and durations of the pheromone stimulus. At any concentration the rising phase of the frequency curve can be fitted by a single exponential function. However, the dynamics of the falling phase depend on the stimulation parameters. For short stimulation periods of 100 and 200 ms at any concentration, the falling phase can be fitted by the sum of two exponential functions, one fast with a small time constant τ_*fall*1_ and one slow with a larger time constant τ_*fall*2_ as shown in Fig 7A. The fitting function used in this case is given by Eq 1. The fitted curves are shown in Fig 7B and the blue (stimulation period: 100 ms, stimulation dose: 10 ng) and purple curves (stimulation period: 200 ms, stimulation dose: 10 ng) in Fig 7D. The fitted falling time constants decrease with pheromone concentration (Table 1). For stimulation concentration at 10 ng with long stimulation periods of 500 ms and 1000 ms, the falling phase of frequency undergoes two stages: a rapid falling stage to a plateau and a slow falling stage. The rapid falling stage can be fitted by one exponential function with a small time constant τ_*fall*1_ and the slow falling stage can be fitted by two exponential functions with one intermediate time constant τ_*fall*2_ and one larger time constant τ_*fall*3_ as shown in Fig 7C. The fitting function used in this case is given by Eq 2. The fitted curves are shown by the green and red curves in Fig 7D. The fitted parameter values of Eqs 1 and 2 are given in Table 1.

$$\bar{f}\left(t\right)=\left\{ \begin{array}{l} f_{sp},\;if\;t\leq t_{sti}+T_{lat}\\ f_{sp}+\left(f_{pe}-f_{sp}\right)\cdot \left(1-e^{-\frac{t-t_{sti}-T_{lat}}{\tau_{rise}}}\right),\;else\;if\left(t_{sti}+T_{lat}\right)\leq t\leq t_{sti}+T_{lat}+T_{d2pe}\\ f_{sp}+\left(f_{pe}-f_{sp}\right)\cdot \left(1-e^{-\frac{T_{d2pe}}{\tau_{rise}}}\right)\cdot \left(qe^{-\frac{t-\left(t_{sti}+T_{lat}+T_{d2pe}\right)}{\tau_{fall1}}}+\left(1-q\right)e^{-\frac{t-\left(t_{sti}+T_{lat}+T_{d2pe}\right)}{\tau_{fall2}}}\right),\;otherwise \end{array}\right.$$ (1)

$$\bar{f}\left(t\right)=\left\{ \begin{array}{l} f_{sp},\;if\;t\leq t_{sti}+T_{lat}\\ f_{sp}+\left(f_{pe}-f_{sp}\right)\cdot \left(1-e^{-\frac{t-t_{sti}-T_{lat}}{\tau_{rise}}}\right),\;else\;if\left(t_{sti}+T_{lat}\right)\leq t\leq t_{pe}\\ f_{pl}+\left(f_{sp}+\left(f_{pe}-f_{sp}\right)\cdot \left(1-e^{-\frac{T_{d2pe}}{\tau_{rise}}}\right)-f_{pl}\right)e^{-\frac{t-\left(t_{sti}+T_{lat}+T_{d2pe}\right)}{\tau_{fall1}}}\;else\;if\;\left(t_{sti}+T_{lat}+T_{d2pe}\right)\leq t\leq \left(t_{sti}+T_{lat}+T_{d2pe}+T_{pl}\right)\\ f_{sp}+\left(f_{pl}-f_{sp}\right)\cdot \left(qe^{-\frac{t-\left(t_{sti}+T_{lat}+T_{d2pe}+T_{pl}\right)}{\tau_{fall2}}}+\left(1-q\right)e^{-\frac{t-\left(t_{sti}+T_{lat}+T_{d2pe}+T_{pl}\right)}{\tau_{fall3}}}\right),\;otherwise \end{array}\right.$$ (2)

where, *f*_*sp*_, *f*_*pe*_ and *f*_*pl*_ are the mean spontaneous frequency of the ORN, peak frequency and plateau frequency in response to stimulation; *t*_*sti*_ the time of stimulation onset; *T*_*lat*_, *T*_*d2pe*_ and *T*_*pl*_ the response latency of the ORN population, the duration to peak frequency from *t*_sti_ + *T*_lat_ and duration of the plateau; τ_*rise*_, τ_*fall1*_, τ_*fall2*_ and τ_*fall3*_ the rising and falling time constants respectively; *q* coefficient of the fast falling component.

We modelled the ORN spike train by a Poisson process characterized by a single parameter, the mean firing rate $\overset{-}{f}(t)$ given by Eqs (1) and (2). For sufficiently short interval *δt*, and a mean frequency $\overset{-}{f}(t)$ varying slowly with respect to *δt*, the probability of a spike occurring during *δt* is equal to the value of the instantaneous firing frequency during this time interval times the length of the interval

$$P\left\{1\;spike\;during\;\delta t\right\}\approx \bar{f}\left(t\right)\cdot \delta t.$$ (3)

At iteration time *t*, a random number *R*[*t*]], uniformly distributed between 0 and 1 is generated. If $R[t]\leq \overset{-}{f}(t)∙\delta t$ where δ*t* is the time step used in simulations, the membrane potential of ORNs is set to *V*_*ORNS*_ = 50 *mV*. Otherwise, *V*_*ORNS*_ = -62 *mV* (resting potential).

### The biophysical model of PN

The model is mathematically described by Hodgkin—Huxley type equations (Eqs (4–14)). The membrane activity of PN satisfies the following differential equation:

$$C_{m}\frac{dV}{dt}=-I_{Na}-I_{Ca}-I_{Kd}-g_{L}\left(V-E_{L}\right)-I_{A}-I_{SK}-I_{nAch},$$ (4)

where *V* is the membrane potential, *C*_m_ the membrane capacitance, g_*L*_ and *E*_L_ the conductance and reversal potential of the leak current, respectively. The values of these passive parameters are given in Table 2.

*The intrinsic currents of PN*. The intrinsic inward (*I*_Na_ and *I*_Ca_) and outward (*I*_A_, *I*_Kd_ and *I*_SK_) ionic currents in PN are described by

$$I_{j}={\bar{g}}_{j}m^{M}h^{N}\left(V-E_{j}\right),$$ (5)

where ${\overset{-}{g}}_{j}$ and *E*_*j*_ are the maximal mean conductance and reversal potential for the ionic current *j*. The values of these two parameters of each current are given in Table 3. *M* = 3, *N* = 1 for *I*_Na_ and *I*_A_; *M* = 1, *N* = 1 for *I*_Ca_; *M* = 3, *N* = 0 for *I*_Kd_; *M* = 2, *N* = 0 for *I*_SK_. The gating variables *m* and *h* in Eq (5) satisfy Eqs (6) and (7) except that *h* = *h*_*∞*_ for *I*_Ca_.

$$\dot{m}=\left(m_{\infty }-m\right)/\tau_{m},$$ (6)

$$\dot{h}=\left(h_{\infty }-h\right)/\tau_{h},$$ (7)

where the steady-state activation *m*_∞_ and inactivation *h*_∞_ of the voltage-activated currents are described by Boltzmann equations as Eqs (8) and (9)

$$m_{\infty }=1/\left\{1+\exp\left[\left(V_{0.5act}-V\right)/S_{m}\right]\right\},$$ (8)

$$h_{\infty }=1/\left\{1+\exp\left[\left(V-V_{0.5inact}\right)/S_{h}\right]\right\}$$ (9)

The voltage dependency of the time constants of *m* and *h* of the voltage-activated currents is described by functions as Eqs (10) and (11) except that the τ_m_ of Ca^2+^ current takes the form of Eq (10’)

$$\tau_{m}\left(V\right)=\frac{1}{a_{\tau m,up}e^{\left(V_{\tau m,0.5up-V}\right)/S_{\tau m,up}}+a_{\tau m,dn}e^{\left(V-V_{\tau m,0.5dn}\right)/S_{\tau m,dn}}},$$ (10)

$$\tau_{m}{}_{Ca}(V)={\left(a_{\left(\tau m,up\right)}exp(\frac{-V}{S_{\tau m,up}})+a_{\left(\tau m,dn\right)}\left(\frac{-V+V_{\tau m,dn}}{\exp\left(\frac{-V+V_{\tau m,dn}}{S_{\tau m,dn}}-1\right)}\right)+\right)}^{-1},$$ (10’)

$$\tau_{h}\left(V\right)=\frac{1}{a_{\tau h,up}e^{\left(V\tau h,0.5up-V\right)/S\tau h,up}+a_{\tau h,dn}e^{\left(V-V\tau h,0.5dn\right)/S\tau h,dn}},$$ (11)

For Na^+^ currents *I*_Na_, value of parameter *V*_0.5act_ in Eq (8) was taken from [38] (DUM cells of the cockroach *Periplaneta americana*), values of parameter *S*_m_, *V*_0.5inact_ and *S*_h_ were modified from [38] and values of parameters in Eqs (10) and (11) were fitted to the data given in [38]. For Ca^2+^ currents *I*_Ca_, values of parameters in Eqs (8) and (9) were taken from [22] (PN in *P*. *americana*), time constant for activation takes the form of Eq (10’) described in [39], *h* = *h*_∞_. For the sustained and transient voltage-gated K^+^ currents *I*_kd_ and *I*_*A*_, values of parameters in Eqs (8) and (9) were taken from [36] (MGC PN in male sphinx moth *Manduca sexta*), and values of parameters in Eqs (10) and (11) were fitted to the data given in [37] (in *M*. *sexta*). The values of various parameters in the voltage dependent steady-state and time constant function of *I*_Na_, *I*_Ca_, *I*_Kd_ and *I*_A_ are given in Table 3.

The mathematical description of *m*_*∞*_ and current of the Ca^2+^-dependent K^+^ currents *I*_SK_ and that of the Ca^2+^ dynamics were borrowed directly from [39] as Eqs (12–14)

$$\frac{dCa}{dt}=-f_{Ca}I_{Ca}-\left(Ca-Ca_{\infty }\right)/\tau_{Ca},$$ (12)

$$m_{S}{}_{K\infty }=1/(1+e^{-a_{m}{}_{sk}-b_{m}{}_{sk}log^{\frac{C_{a}-C_{a\infty }}{S_{msk}}}}),$$ (13)

$$I_{S}{}_{K}={\bar{g}}_{S}{}_{K}\cdot m_{SK\infty }^{2}(V-E_{K}),$$ (14)

where the values of parameters of Ca^2+^ dynamics are given in Table 2 and those of *I*_SK_ are given in Table 3.

*The cholinergic synaptic current from ORN*s *to PN*. The fast nicotinic cholinergic synaptic currents calculated according to

$$I_{nACh}=\sum_{i=1}^{N}{\bar{g}}_{nACh}\cdot {\left[O\right]}_{i}\left(t\right)\cdot \left(V-E_{nACh}\right),$$ (15)

where *N* is the number of ORNs,${\overset{-}{\;g}}_{nACh}$ the mean peak conductance, and *E*_*nAch*_ = 0 *mV* the reversal potential of the current respectively. The fraction of open channels [*O*]_*i*_ is modeled by first-order activation scheme (see review in [40])

$$\frac{d{\left[O\right]}_{i}}{dt}=\alpha \left(1-{\left[O\right]}_{i}\right){\left[T\right]}_{i}-\beta {\left[O\right]}_{i}$$ (16)

The release of cholinergic transmitter [*T*]_*i*_ from *i*th ORN was modeled by a square pulse

$${\left[T\right]}_{i}=A\theta \left(t_{0}+t_{max}-t\right)\theta \left(t-t_{0}\right)$$ (17)

Parameter values of the nACh synaptic current are given in Table 4.

### The MGC network model

We constructed a MGC network model by connecting 100 ORNs to one PN as shown in S1 Fig. No LNs were included in the MGC model in order to test the hypothesis that the triphasic firing patterns of PN can be generated by the ionic currents in PN and ORN inputs. Computer simulations of the model were performed in Microsoft visual studio 2008. The simulation results were analyzed with Matlab 7.5. The total computer simulation time is 25s and the pheromone stimulation started at 5s.

### Low-pass and high-pass Butterworth filters

In order to better understand how different ionic currents contribute to the generation of the PN firing pattern we separated the slow and fast components of the depolarizing and repolarizing currents in PN. We designed 10th-order lowpass and highpass Butterworth filters with cut-off frequency 5 Hz using the Matlab function "butter". By applying the designed lowpass and highpass filters the slow and fast components of each ionic currents were extracted.

### Analysis of the PN response characteristics

The PN response pattern is quantitatively characterized by duration of E_1_ and I phases and frequency of E_1_ and E_2_ phases. These features were defined in S9 Fig and were calculated as follows and expressed as means ± standard error of the mean.

#### Duration

The E_1_ durations were measured from the first spike of the PN response to the spike just preceding the inhibitory phase. The I durations were measured from the last spike of E_1_ to the first spike of E_2_.

#### Frequency

We first calculated the interspike intervals (ISIs) between successive spikes. Then the ISIs were averaged in 10 spikes around each spike using Matlab function smooth. The frequencies are the inverse of the ISIs.

## Supporting Information

S1 Fig Simplified model of moth MGC. The model is composed of 100 Poisson ORNs and one biophysical PN. ORNs receive pheromone stimuli and the PN receive Ach synaptic inputs from ORNs through nicotinic receptors at the dendrites.
(TIF)

S2 Fig Effects of parameters for *m*_∞_, τ_m_ function of *I*_Ca_ and for m_sk∞_ function of *I*_SK_ on PN response duration. (TIF)

S3 Fig Effects of parameters for τ_m_, τ_h_ and *h*_∞_ function of *I*_Na_ on PN response characteristics. I duration clearly increases with *S*_*τ*m,up_ (A) and *V*_*τh*_,_0.5dn_ (G), while it decreases with *a*_*τh*_,_dn_ (C). E_1_ frequency linearly decreases with *V*_0.5inact_ (F); E_2_ frequency increases with *a*_*τ*h,dn_ (D), *S*_*τ*h,dn_ (B) and *V*_*τh*_,_0.5up_ (E) while it decreases with *V*_0.5inact_ (F) and *V*_*τh*_,_0.5dn_ (H).
(TIF)

S4 FigEffects of parameters of *I*_Kd_ on PN response characteristics.
*I*_Kd_ clearly affects E_1_ frequency which increases with $\overset{-}{g}$_Kd_ when $\overset{-}{g}$_Kd_ is below 0.7 μS then decreases (B) while it decreases with *V*_0.5act_ (E) and *S*_m_ of *I*_Kd_ (H).(TIF)

S5 Fig Effects of Ach pulse duration *t*_max_ and concentration *A* on PN response characteristics. Top panel: effects of *t*_max_ on E_1_ and *I* duration (A) and mean firing frequency of E_1_ and E_2_ (B). Bottom panel: effects of *A* on E_1_ and *I* duration (C) and mean firing frequency of E_1_ and E_2_ phases (D).
(TIF)

S6 FigEffects of nAch postsynaptic channel opening rate α, closing rate β and $\overset{-}{g}$_nACh_ on PN response characteristics.Top panel: effects of *α* on E_1_ and I duration (A) and mean firing frequency of E_1_ and E_2_ (B). Middle panel: effects of *β* on E_1_ and I duration (C) and mean firing frequency of E_1_ and E_2_ phases (D). Bottom panel: effects of $\overset{-}{g}$_nACh_ on E_1_ and I duration (E) and mean firing frequency of E_1_ and E_2_ (F).(TIF)

S7 Fig Effects of inhibition mediated by fast GABA_A_ receptors on the activity patterns of PN and three LNIs in a network with 80 ORNs, 1 PN and 20 LNIs. The postsynaptic channel closing rate β of the GABA synapses from LNI to PN is 0.1 (left panel), 2.0 (middle panel) and 3.0 (right panel) respectively.
(TIF)

S8 Fig Effects of slow inhibition mediated by metabotropic GABA_B_ receptors on the response activity of PN and three LNIs in a network with 80 ORNs, 1 PN and 20 LNIs. Panel A-D show that the GABA_B_ mediated synaptic inhibition did not alter the triphasic response pattern of PN in the normal parameter range: A. PN potential, spikes and frequency; B. potentials of LNI8; C. normalized concentration of intracellular Ca in PN and of GABA_B_ receptor-coupled G protein; D. potentials of LNI17. Panel E-F show that the I duration is prolonged when *r*_3_ is decreased: E. PN potential, spikes and frequency; F. normalized concentration of intracellular Ca in PN and of GABA_B_ receptor-coupled G protein. Panel G-H show that the GABA_B_ mediated synaptic inhibition changed the triphasic response pattern when *r*_3_ is increased and *r*_4_ is decreased: G. PN potential, spikes and frequency; H. normalized concentration of intracellular Ca in PN and of GABA_B_ receptor-coupled G protein.
(TIF)

S9 Fig Extracellularly recorded response patterns of the moth pheromone sensitive PN in MGC. Left panel: top trace, response pattern to low dose pheromone stimulus; bottom trace, response pattern to high dose pheromone stimulus. Right panel: from top to bottom trace the duration of pheromone stimuli were increased at a given stimulation concentration.
(TIF)

S1 Text Model of type I LNs with sodium spikes. (PDF)

S2 Text Experimental findings in ORNs, PNs and type I LNs. (PDF)
