# Supplementary figures and images for: Modeling the Cellular Mechanisms and Olfactory Input Underlying the Triphasic Response of Moth Pheromone-Sensitive Projection Neurons
Source: PLoS One. 2015 May 11;10(5):e0126305. doi: 10.1371/journal.pone.0126305 (PMC4427114; doi:10.1371/journal.pone.0126305)

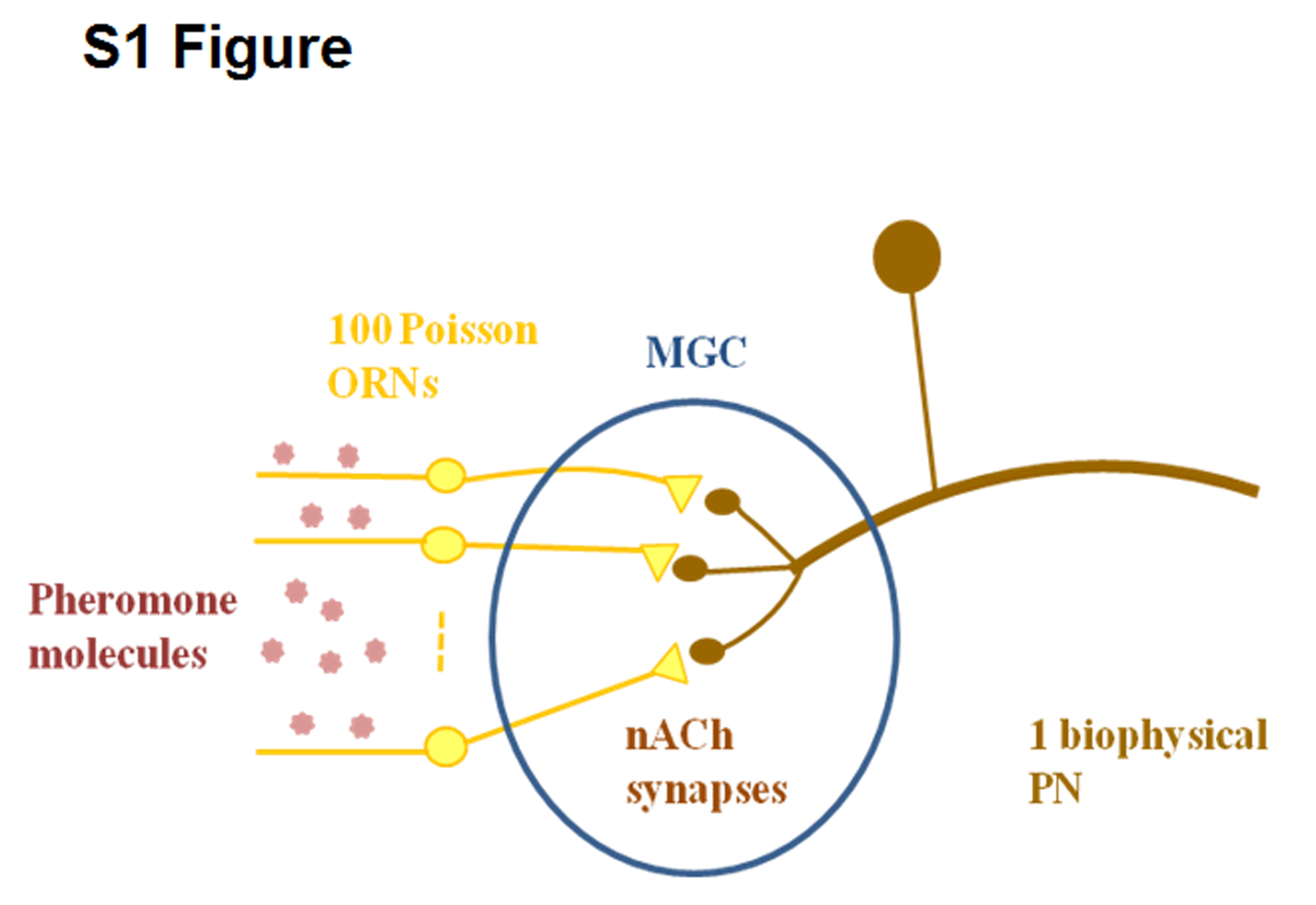

Supplement: S1 Fig — The model is composed of 100 Poisson ORNs and one biophysical PN. ORNs receive pheromone stimuli and the PN receive Ach synaptic inputs from ORNs through nicotinic receptors at the dendrites. (TIF) [file pone.0126305.s001.tif]

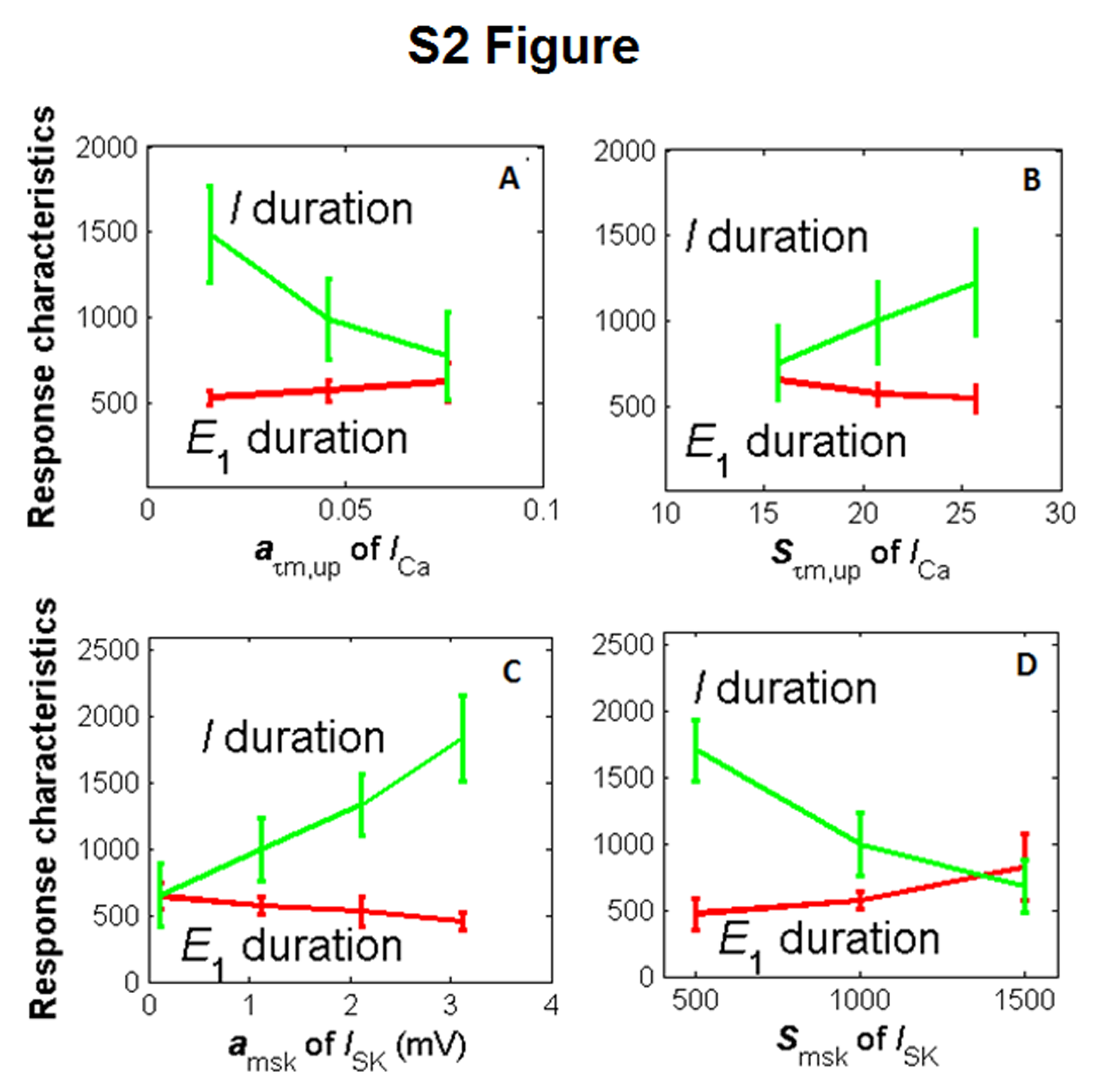

Supplement: S2 Fig — (TIF) [file pone.0126305.s002.tif]

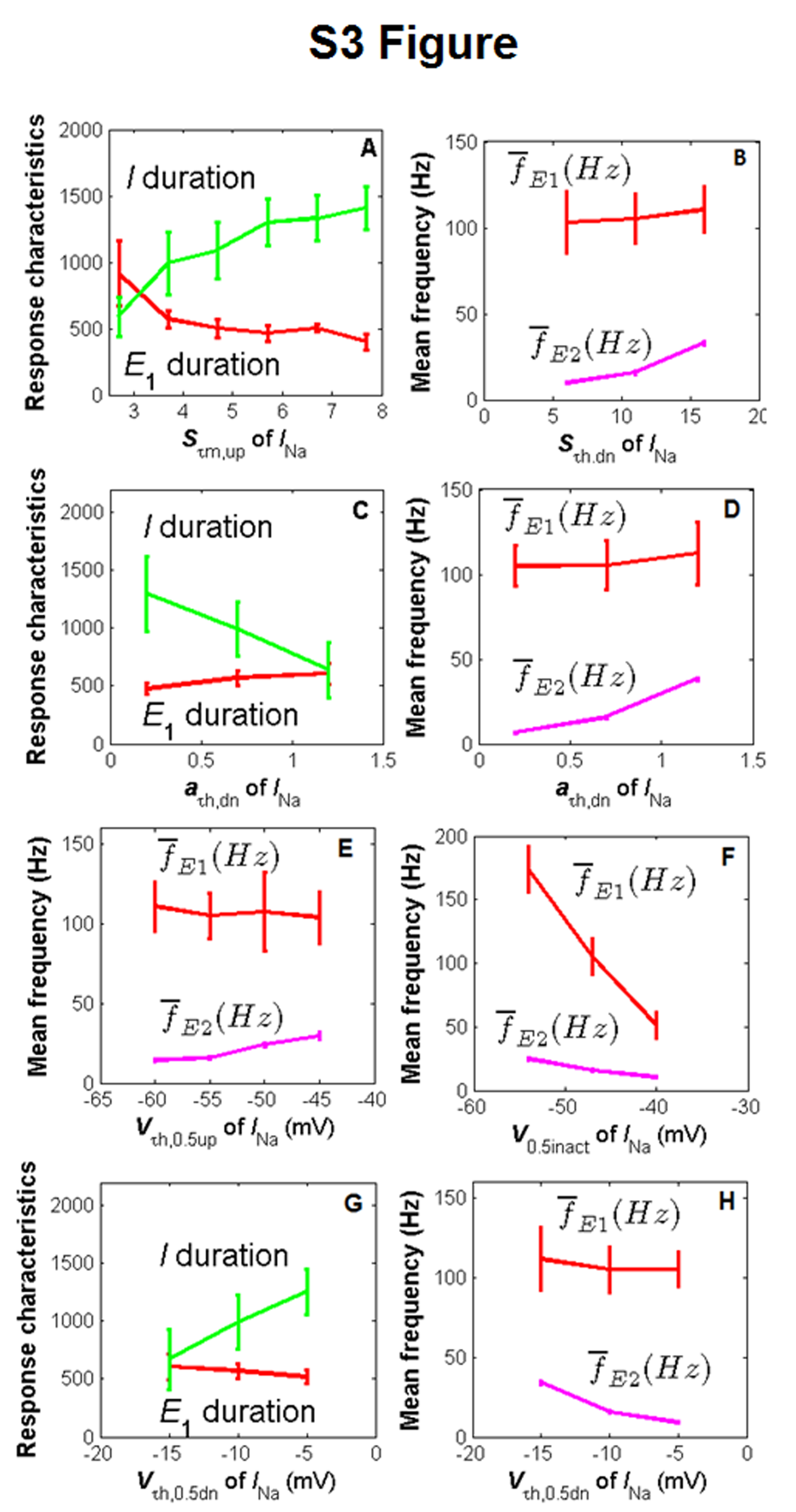

Supplement: S3 Fig — I duration clearly increases with S τm,up (A) and V τh,0.5dn (G), while it decreases with a τh,dn (C). E1 frequency linearly decreases with V 0.5inact (F); E2 frequency increases with a τh,dn (D), S τh,dn (B) and V τh,0.5up (E) while it decreases with V 0.5inact (F) and V τh,0.5dn (H). (TIF) [file pone.0126305.s003.tif]

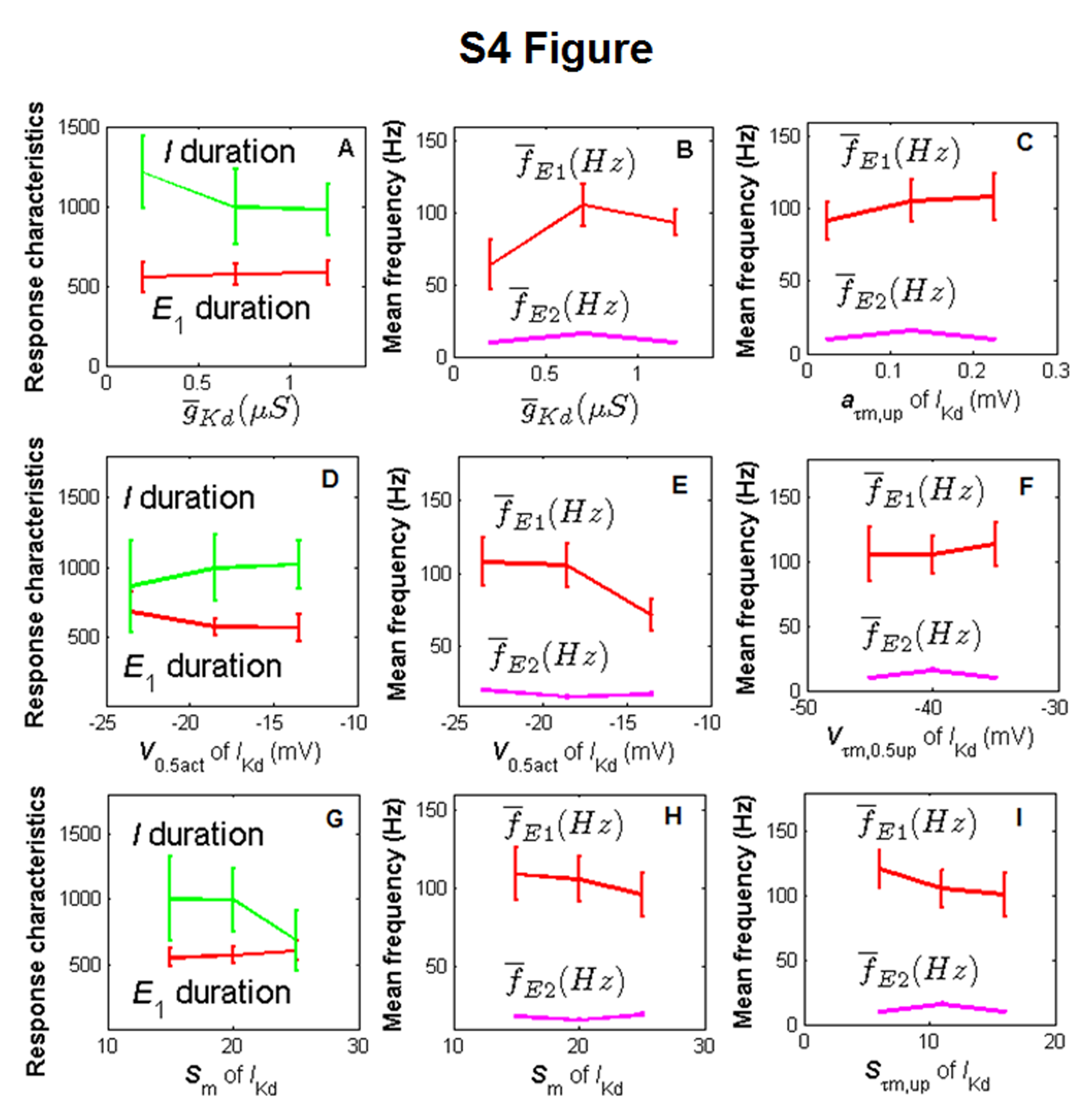

Supplement: S4 Fig — I Kd clearly affects E1 frequency which increases with g- Kd when g- Kd is below 0.7 μS then decreases (B) while it decreases with V 0.5act (E) and S m of I Kd (H). (TIF) [file pone.0126305.s004.tif]

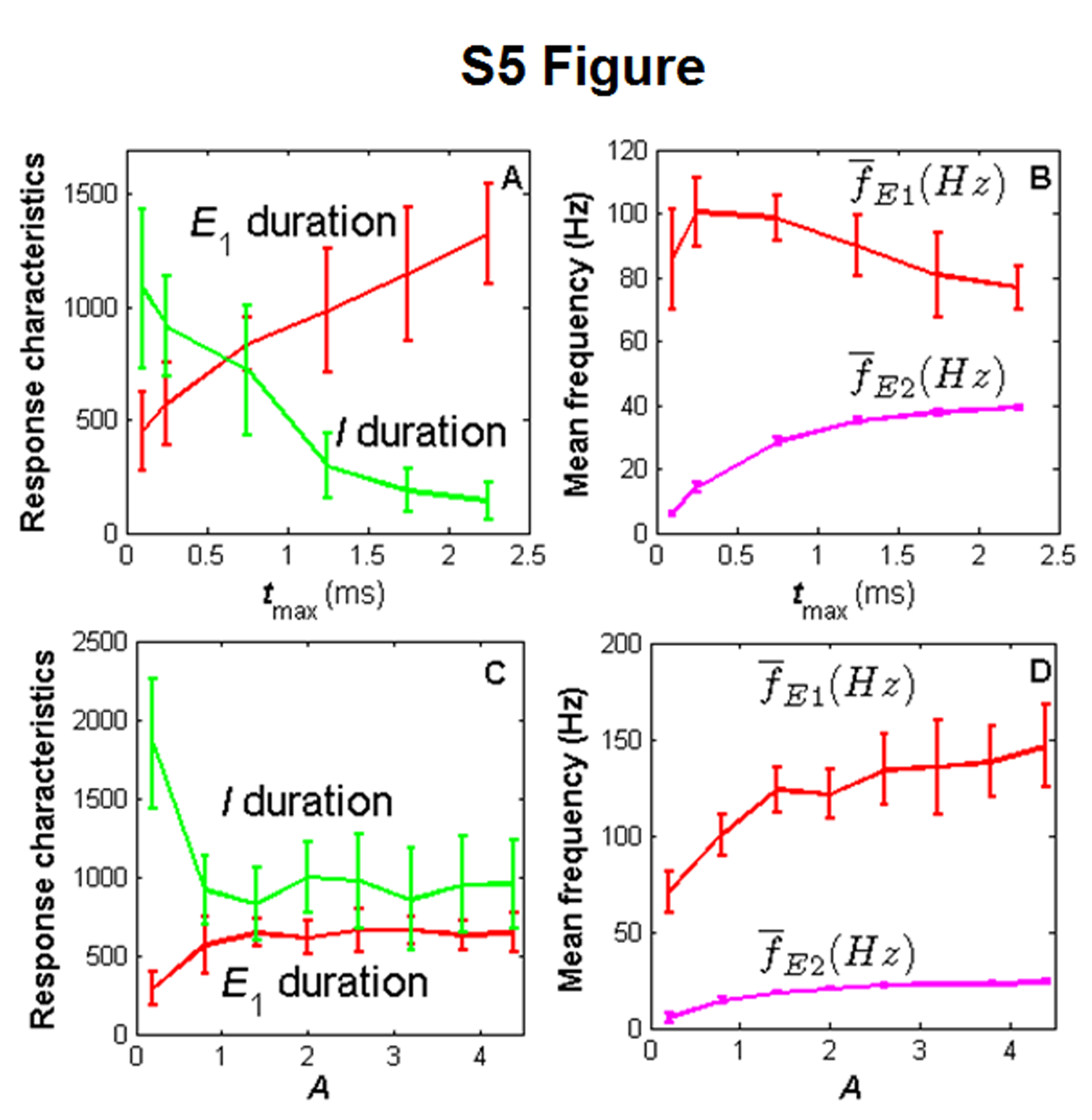

Supplement: S5 Fig — Top panel: effects of t max on E1 and I duration (A) and mean firing frequency of E1 and E2 (B). Bottom panel: effects of A on E1 and I duration (C) and mean firing frequency of E1 and E2 phases (D). (TIF) [file pone.0126305.s005.tif]

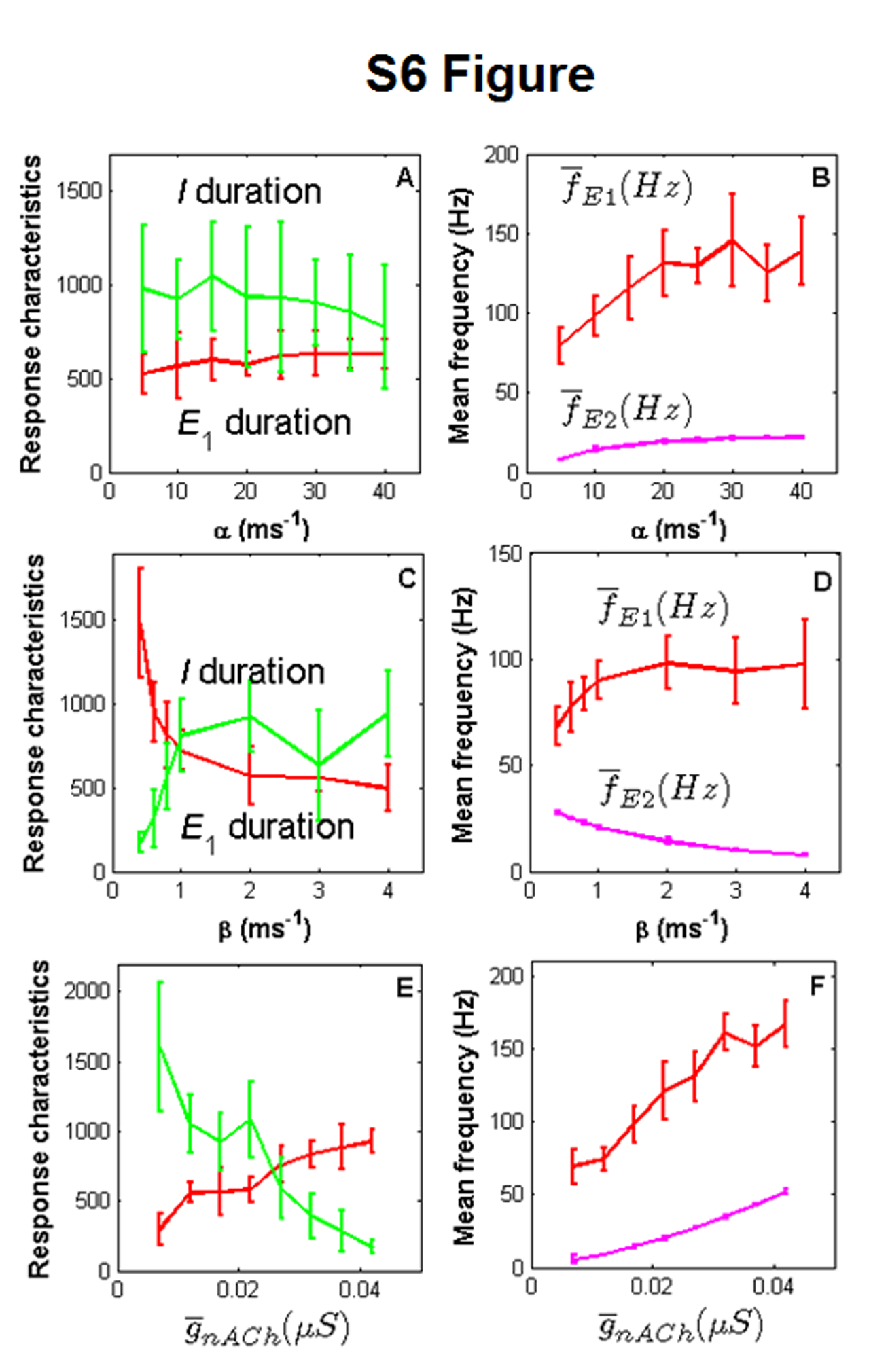

Supplement: S6 Fig — Top panel: effects of α on E1 and I duration (A) and mean firing frequency of E1 and E2 (B). Middle panel: effects of β on E1 and I duration (C) and mean firing frequency of E1 and E2 phases (D). Bottom panel: effects of g- nACh on E1 and I duration (E) and mean firing frequency of E1 and E2 (F). (TIF) [file pone.0126305.s006.tif]

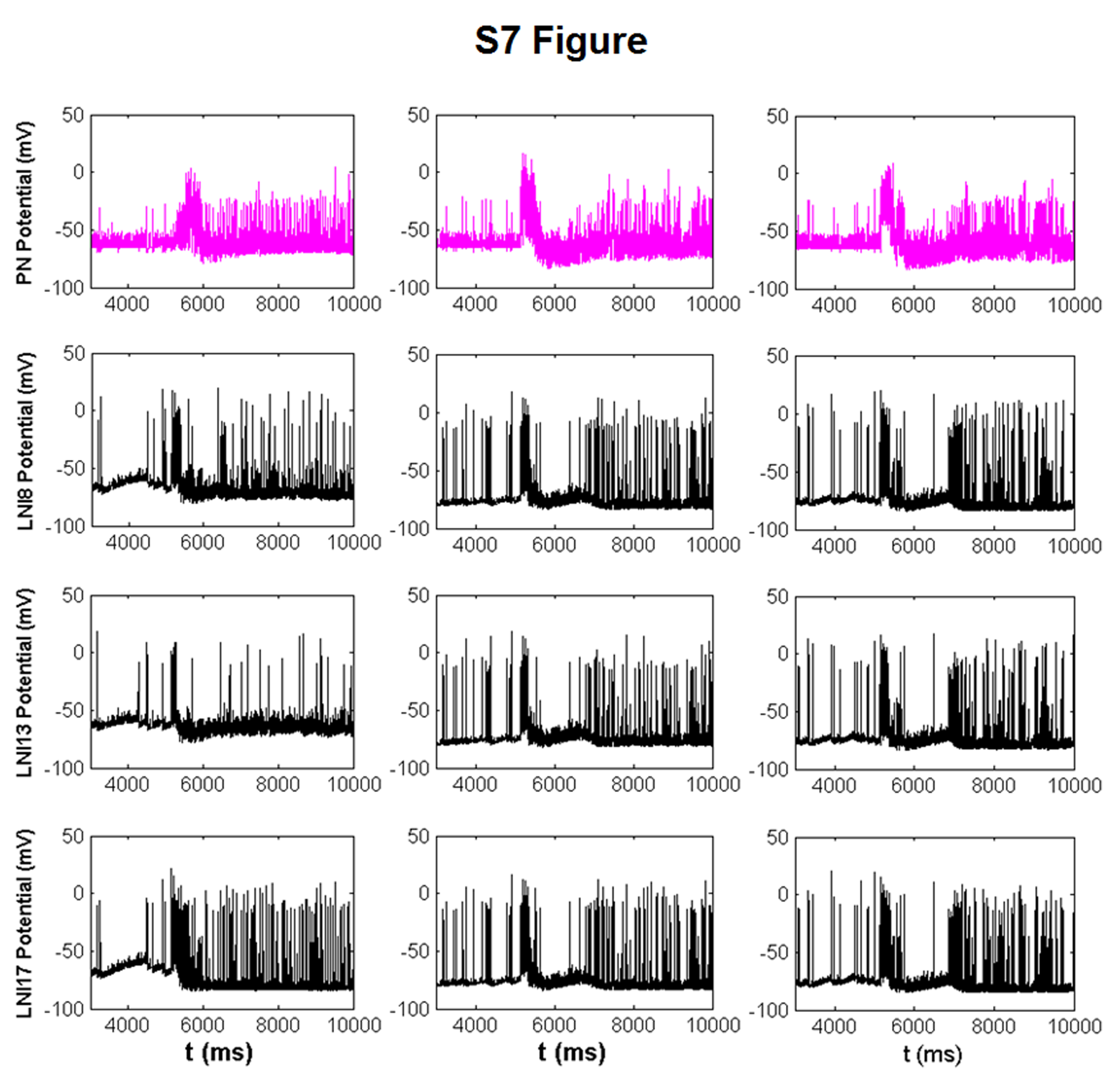

Supplement: S7 Fig — The postsynaptic channel closing rate β of the GABA synapses from LNI to PN is 0.1 (left panel), 2.0 (middle panel) and 3.0 (right panel) respectively. (TIF) [file pone.0126305.s007.tif]

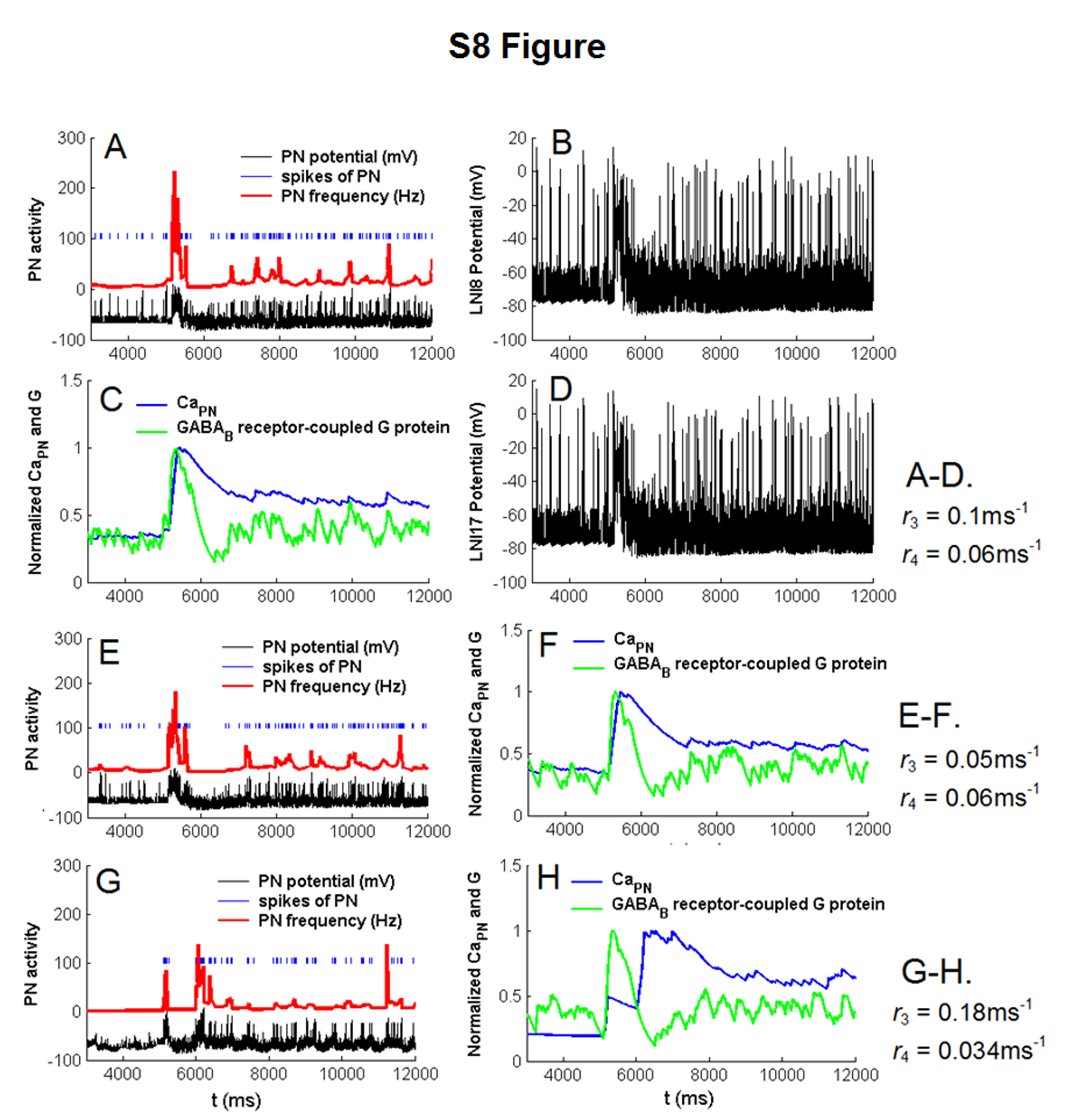

Supplement: S8 Fig — Panel A-D show that the GABAB mediated synaptic inhibition did not alter the triphasic response pattern of PN in the normal parameter range: A. PN potential, spikes and frequency; B. potentials of LNI8; C. normalized concentration of intracellular Ca in PN and of GABAB receptor-coupled G protein; D. potentials of LNI17. Panel E-F show that the I duration is prolonged when r 3 is decreased: E. PN potential, spikes and frequency; F. normalized concentration of intracellular Ca in PN and of GABAB receptor-coupled G protein. Panel G-H show that the GABAB mediated synaptic inhibition changed the triphasic response pattern when r 3 is increased and r 4 is decreased: G. PN potential, spikes and frequency; H. normalized concentration of intracellular Ca in PN and of GABAB receptor-coupled G protein. (TIF) [file pone.0126305.s008.tif]

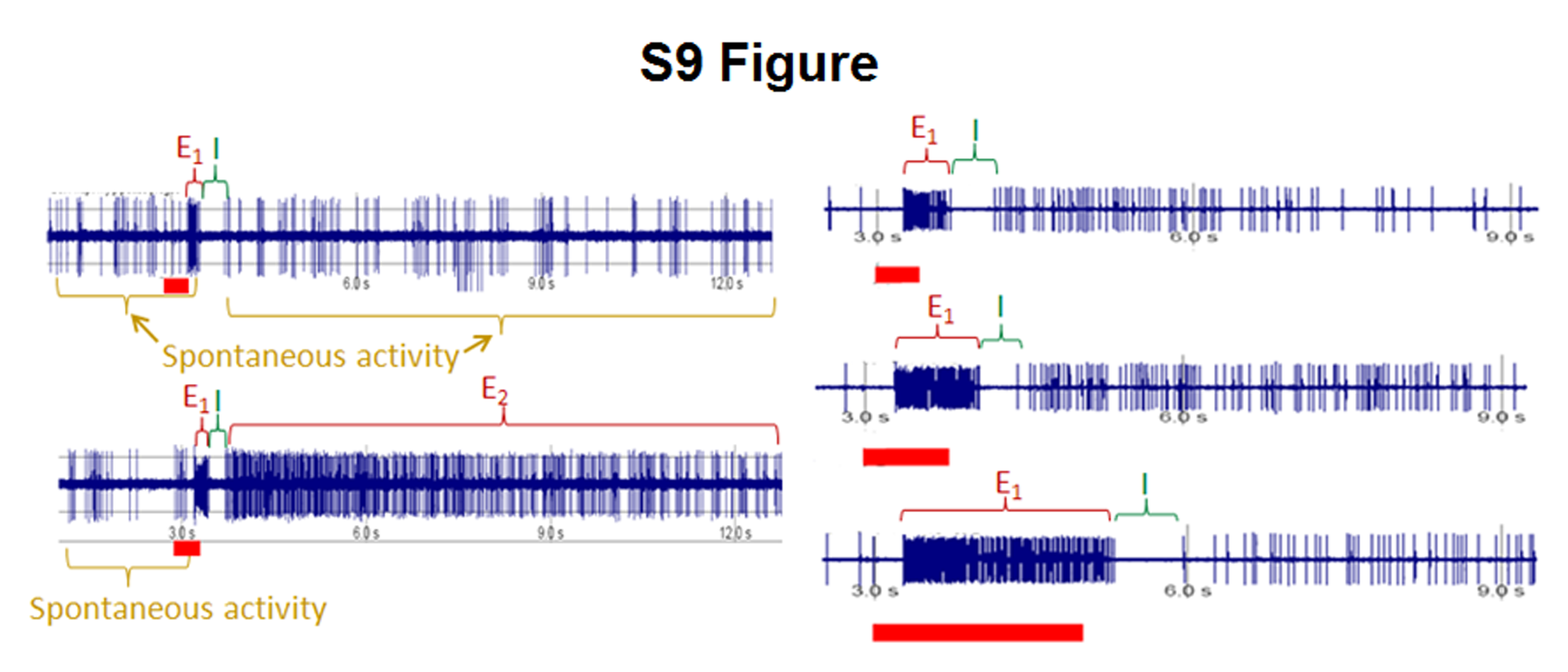

Supplement: S9 Fig — Left panel: top trace, response pattern to low dose pheromone stimulus; bottom trace, response pattern to high dose pheromone stimulus. Right panel: from top to bottom trace the duration of pheromone stimuli were increased at a given stimulation concentration. (TIF) [file pone.0126305.s009.tif]
