# Supplementary material for: Modeling the Cellular Mechanisms and Olfactory Input Underlying the Triphasic Response of Moth Pheromone-Sensitive Projection Neurons
Source: PLoS One. 2015 May 11;10(5):e0126305. doi: 10.1371/journal.pone.0126305 (PMC4427114; doi:10.1371/journal.pone.0126305)
Supplement: S1 Text — (PDF) [file pone.0126305.s010.pdf]

S1_Text. Model of type I LNs with sodium spikes

We reconstruct the LNI by the following equation

$$C_m \frac{dV}{dt} = -I_{Na} - I_{Ca} - I_{Kd} - g_L(V - E_L) - I_A - I_{K(Ca)} - I_{nACh}, \qquad \text{(S1)}$$

where, the intrinsic ionic currents, gating variables and their steady-state activation and inactivation are described by Eq. (5) to (9); The voltage dependency of the time constants of m and h of the voltage-activated currents is described by functions as Eqs. (10) and (11) in the main text except that the τ_m of Ca^{2+} current takes the form of Eq. (S2) (Laurent, et al., 1993)

$$\tau_{mCa}(V) = 1 + 0.014(V + 30). \qquad \text{(S2)}$$

The values of various parameters of the LNI model are given in Table S1. The cholinergic synaptic currents from ORNs and PN to LNI and the GABAergic synaptic currents mediated by fast $GABA_A$ receptors from LNI to PN are described by Eq. (15) to (17) in the main text. Where reversal potential E_{GABAA} = -70 mV, t_{max} = 0.3 ms, A = 0.8 and α = 1.0 ms^{-1}. The slow GABAergic synaptic currents mediated by metabotropic $GABA_B$ receptors from LNI to PN are given by Eq. (S3) (Destexhe et al., 1996; Bazhenov et al., 1998)

$$I_{slow-inh} = \bar{g}_{slow-inh} \frac{[G]^4}{[G]^4 + K} (V - E_{GABA-slow}),$$

$$\frac{d[R]}{dt} = r_1(1 - [R])[T] - r_2[R], \qquad \text{(S3)}$$

$$\frac{d[G]}{dt} = r_3[R] - r_4[G],$$

where $E_{GABA\text{-}slow}$ = -91.6 mV is the potassium reversal potential, $[R]$ the fraction of activated receptors, $[G]$ the concentration of G proteins, The rate constants are $r_1 = 1.0\ mM^{-1}ms^{-1}$, $r_2 = 0.0025\ ms^{-1}$, $r_3 = 0.1ms^{-1}$, $r_4 = 0.06ms^{-1}$, and $K = 100\ \mu M^4$.

Table S1. Parameter values of the LNI model given or calculated from patch-clamp data

<table>
<tr><td rowspan="2">Passive parameter values</td><td colspan="2">C_m(pF)</td><td>E_L (mV)</td><td>g_L (nS)</td><td colspan="3">Ref.</td></tr>
<tr><td colspan="2">36.8</td><td>-53.0</td><td>19.76[a]</td><td colspan="3">Husch et al., 2009</td></tr>
<tr><td rowspan="2">Steady-state functions of I_{Na}</td><td>$\bar{g}_{Na}$(nS)</td><td>$V_{0.5act}$ (mV)</td><td>S_m</td><td>$V_{0.5inact}$ (mV)</td><td>S_h</td><td>E_{Na} (mV)</td><td>Ref.</td></tr>
<tr><td>206 modified 2500</td><td>-25.8</td><td>9.32 modified 11</td><td>-41.1 modified 50.34</td><td>9.75 modified 5.5</td><td>+48.2(RP) +47.9(EP)</td><td>Lapied et al., 1990</td></tr>
<tr><td rowspan="4">Time constant functions of I_{Na}</td><td>$a_{\tau m,up}$</td><td>$V_{\tau m,0.5up}$ (mV)</td><td>$S_{\tau m,up}$</td><td>$a_{\tau m,dn}$</td><td>$V_{\tau m,0.5dn}$</td><td>$S_{\tau m,dn}$</td><td>Ref.</td></tr>
<tr><td>0.5</td><td>-30</td><td>3.7</td><td>0.5</td><td>-15</td><td>13.7</td><td rowspan="3">Fitted to Lapied et al., 1990</td></tr>
<tr><td>$a_{\tau h,up}$</td><td>$V_{\tau h,0.5up}$</td><td>$S_{\tau h,up}$</td><td>$a_{\tau h,dn}$</td><td>$V_{\tau h,0.5dn}$</td><td>$S_{\tau h,dn}$</td></tr>
<tr><td>2.1</td><td>-55</td><td>5</td><td>0.7</td><td>-10</td><td>11</td></tr>
</table>

Steady-state functions of I_{Ca}	$\bar{g}_{Ca}$(nS)	$V_{0.5act}$ (mV)	S_m	$V_{0.5inact}$ (mV)	S_h	E_{Ca}(mV)	Ref.
	10.4 modified 100	-10.6 modified -11.6	8.5 modified 10.0	-29.6 modified -30	8.4 modified 18.3	160	Husch et al., 2009
Time constant functions of I_{Ca}	$a_{\tau h,up}$	$S_{\tau h,up}$	$a_{\tau h,dn}$	$V_{\tau h,dn}$	$S_{\tau h,dn}$	--	Ref.
	0.002	29	0.3	40	13	--	Laurent, et al., 1993
Ca ynamics	Ca_{∞}(nM)	τ_{Ca} (ms)		f_{Ca} --		--	Ref.
	113.0	656 modified 2000		1.6 modified 1.0		--	Roper et al., 2003
Steady-state function of $I_{k(Ca)}$	a_{msk}	b_{msk}		S_{msk}	--	--	Ref.
	1.120	2.508		1000	--	--	Roper et al., 2003
Steady tate function of I_{Kd}	$\bar{g}_{Kd}$ (nS)	$V_{0.5act}$ (mV)		S_m	E_K (mV)		Ref.
	8.17[b] modified 1000	-18.5		22.5 modified 20.0	-91.6	--	Kloppenburg et al., 1999
Time constant functions of I_{Kd}	$a_{\tau m,up}$	$V_{\tau m,0.5up}$		$S_{\tau m,up}$	--	--	Ref.
	0.125	-40		11.0	--	--	Fitted to Mercer et al.,1995
	$a_{\tau m,dn}$	$V_{\tau m,0.5dn}$		$S_{\tau m,dn}$	--	--	
	0.15	25		45.7	--	--	
Steady-state functions of I_A	$\bar{g}_A$ (nS)	$V_{0.5act}$ (mV)	S_m	$V_{0.5inact}$ (mV)	S_h	E_K(mV)	Ref.
	17.35[c] modified 600	-32.69 modified -9	17.5	-53.3	7.23	-91.6	Kloppenburg et al., 1999
Time constant functions of I_A	$a_{\tau m,up}$	$V_{\tau m,0.5up}$	$S_{\tau m,up}$	$a_{\tau m,dn}$	$V_{\tau m,0.5dn}$	$S_{\tau m,dn}$	Fitted to Mercer et al., 1995
	0.5	-30	13.7	0.42	-15	46	
	$a_{\tau h,up}$	$V_{\tau h,0.5up}$	$S_{\tau h,up}$	$a_{\tau h,dn}$	$V_{\tau h,0.5dn}$	$S_{\tau h,dn}$	
	0.04	-55	25	0.045	40	55	

[a]Calculated by $g_L = 1/R_M = 1/50.6\ M\Omega = 0.01976\ \mu S = 19.76$ nS.
[b,c]Calculated from Kloppenburg et al.,1999.

References

Bazhenov M, Timofeev I, Steriade M, and Sejnowski TJ (1998) Cellular and network models for intrathalamic augmenting responses during 10 Hz stimulation. J Neurophysiol 79: 2730–2748.

Destexhe A, Bal T, McCormick DA, and Sejnowski TJ (1996) Ionic mechanisms underlying synchronized oscillations and propa- gating waves in a model of ferret thalamic slices. J Neurophysiol 76: 2049–2070.

Husch A, Paehler M, Fusca D, Paeger L, and Kloppenburg P (2009) Calcium current diversity in physiologically different local interneuron types of the antennal lobe. J Neurosci 29(3):716-726.

Lapied B, Malecot CO, Pelhate M (1990) Patch-clamp study of the properties of the sodium current in cockroach single isolated adult aminergic neurons. J Exp Biol 151: 387–404.

Laurent G, Seymour-Laurent KJ, and Johnson K (1993) Dendritic excitability and a voltage-gated calcium current in locust nonspiking local interneurons. J Neurophysiol 69: 1484–1498.

Mercer AR, Hayashi JH, Hildebrand JG (1995) Modulatory effects of 5-hydroxytryptamine on voltage-activated currents in cultured antennal lobe neurons of the sphinx moth *Manduca sexta*. J Exp Biol 198: 613–627.

Roper P, Callaway J, Shevchenko T, Teruyama R, Armstrong W (2003) AHP's, HAP's and DAP's: How potassium currents regulate the excitability of rat supraoptic neurones. J Comput Neurosci 15: 367-389.

Kloppenburg P, Ferns D, Mercer AR (1999) Serotonin enhances central olfactory neuron responses to female sex pheromone in the male sphinx moth *Manduca sexta*. J Neurosci 19(19): 8172–8181.
