# Supplementary material for: Modeling the Cellular Mechanisms and Olfactory Input Underlying the Triphasic Response of Moth Pheromone-Sensitive Projection Neurons
Source: PLoS One. 2015 May 11;10(5):e0126305. doi: 10.1371/journal.pone.0126305 (PMC4427114; doi:10.1371/journal.pone.0126305)
Supplement: S2 Text — (PDF) [file pone.0126305.s011.pdf]

S2_Text. Experimental findings in ORNs, PNs and type I LNs

We summarize the main response characteristics of ORNs and PNs in moth MGC based on the intra- and extracellular recorded data and the main ionic currents in MGC PNs and type I LNs in moths and other insects based on patch-clamp data.

Response patterns of the moth pheromone sensitive PNs in MGC. PNs responses to pheromone stimuli were recorded extracellularly from the MGC of the moth *Agrotis ipsilon* (Martinez et al 2013). At low concentrations, the response of most pheromone sensitive PNs is biphasic and consists of an excitatory phase followed by an inhibitory phase as shown in the top trace on the left panel of S9_Fig. At higher concentrations the response becomes triphasic with a long tonic excitatory phase occurring after the inhibition phase (bottom trace on the left panel of S9_Fig.). So, four typical firing phases can be distinguished − the slow spontaneous activity before stimulation, the tonic firing phase induced by pheromone stimulus (denoted E_1), the inhibitory period just after the end of the pheromone stimulus (denoted I) and a second excitation phase (denoted E_2). When the duration of the pheromone stimulus is increased, the duration of the E_1 phase increases correspondingly whereas the inhibitory phase remains almost constant (right panel of S9_Fig.). The firing rate during E_1 shows a clear adaptation.

Comparison of response characteristic of ORNs with that of PNs to the same pheromone stimulus. In order to find out whether the triphasic response pattern is generated in ORNs or in PNs, simultaneous recordings from both ORNs and PNs were carried out (Jarriault et al., 2010). The bottom trace in Fig. 1A and black curve of Fig. 1C in Jarriault et al., 2010 show typical triphasic response characteristics in PNs. However, no quiescent period was observed in the recorded ORNs as shown in the top trace in Fig. 1A and the gray curve in Fig. 1C in Jarriault et al., 2010. These results demonstrate that the PN triphasic response pattern is generated intrinsically in PNs and/or in the MGC network.

Whole-cell patch-clamp data on pheromone sensitive PNs, on the other types of neurons and synapses in insects. To better understand the mechanisms underlying the PN firing pattern, we seek for the intrinsic types and characteristics of ionic channels presenting in PN. Using whole cell patch-clamp technique on PNs in cockroach (Husch et al., 2009) and in moth (Mercer and Hildebrand, 2002), five main components of the inward and outward currents were identified. Two inward currents are the fast activating/inactivating Na^+ current (I_{Na}) and the smaller, slowly inactivating inward Ca^{2+} current (I_{Ca}). Three outward currents are the transient, voltage-dependent K^+ current (I_A), the sustained, voltage-dependent K^+ current (I_{Kd}) and the Ca^{2+}-dependent, outward current K^+ ($I_{K(Ca)}$).

To further study the possible influence of LNs on the response characteristics of PN, we attempt to reconstruct a model of LNs based on their ionic currents found by experiments. One type of LNs with inward sodium currents I_{Na} and other types of currents such as I_{Ca}, I_{Kd}, $I_{K(Ca)}$ and I_A, which was named as type LNI in Husch et al., 2009, have been identified by patch-clamp experiments in moth (Mercer and Hildebrand, 2002) and in cockroach (Husch et al., 2009). It was also found that LNIs are GABAergic. In a recent work, Warren and Kloppenburg (2014) have demonstrated functionnally the existence of fast and slow GABAergic synapses ($GABA_A$ and $GABA_B$) from LNs onto PNs and cholinergic synapses from from PNs onto LNs in the cockroach Periplaneta americana.

References

Husch A, Paehler M, Fusca D, Paeger L, and Kloppenburg P (2009) Calcium current diversity in physiologically different local interneuron types of the antennal lobe. J Neurosci 29(3):716-726.

Jarriault D, Gadenne C, Lucas P, Rospars J-P, Anton S (2010) Transformation of the sex pheromone signal in the noctuid moth *Agrotis ipsilon*: from peripheral input to antennal lobe output. Chem Senses 35: 705–715.

Martinez D, Chaffiol A, Voges N, Gu Y, Anton S, Rospars J-P, Lucas P (2013) Multiphasic On/Off Pheromone Signalling in Moths as Neural Correlates of a Search Strategy. PLoS ONE 8(4): e61220. doi:10.1371/journal.pone.0061220.

Mercer AR and Hildebrand JG (2002) Developmentalchanges in the density of ionic currents in antennal-lobeneurons of the sphinx moth, *Manducasexta*.J Neurophysiol87: 2664–2675.

Warren B, Kloppenburg P (2014) Rapid and slow chemical synaptic interactions of cholinergic projection neurons and GABAergic local interneurons in the insect antennal lobe. J Neurosci 34: 13039-13046.
